# Comprehensive analysis of bulk and single-cell transcriptomic data reveals a novel signature associated with endoplasmic reticulum stress, lipid metabolism, and liver metastasis in pancreatic cancer

**DOI:** 10.1186/s12967-024-05158-y

**Published:** 2024-04-29

**Authors:** Xiaohong Liu, Bo Ren, Yuan Fang, Jie Ren, Xing Wang, Minzhi Gu, Feihan Zhou, Ruiling Xiao, Xiyuan Luo, Lei You, Yupei Zhao

**Affiliations:** 1 Department of General Surgery, Peking Union Medical College Hospital, Peking Union Medical College, Chinese Academy of Medical Sciences, Beijing, 100023 People’s Republic of China; 2 Key Laboratory of Research in Pancreatic Tumor, Chinese Academy of Medical Sciences, Beijing, 100023 People’s Republic of China; 3 National Science and Technology Key Infrastructure On Translational Medicine in Peking Union Medical College Hospital, Beijing, 100023 People’s Republic of China

**Keywords:** Pancreatic cancer, Endoplasmic reticulum stress, Lipid metabolism, Immune environment, Cell–cell communication

## Abstract

**Background:**

Pancreatic ductal adenocarcinoma (PDAC) is a lethal malignancy with high probability of recurrence and distant metastasis. Liver metastasis is the predominant metastatic mode developed in most pancreatic cancer cases, which seriously affects the overall survival rate of patients. Abnormally activated endoplasmic reticulum stress and lipid metabolism reprogramming are closely related to tumor growth and metastasis. This study aims to construct a prognostic model based on endoplasmic reticulum stress and lipid metabolism for pancreatic cancer, and further explore its correlation with tumor immunity and the possibility of immunotherapy.

**Methods:**

Transcriptomic and clinical data are acquired from TCGA, ICGC, and GEO databases. Potential prognostic genes were screened by consistent clustering and WGCNA methods, and the whole cohort was randomly divided into training and testing groups. The prognostic model was constructed by machine learning method in the training cohort and verified in the test, TCGA and ICGC cohorts. The clinical application of this model and its relationship with tumor immunity were analyzed, and the relationship between endoplasmic reticulum stress and intercellular communication was further explored.

**Results:**

A total of 92 characteristic genes related to endoplasmic reticulum stress, lipid metabolism and liver metastasis were identified in pancreatic cancer. We established and validated a prognostic model for pancreatic cancer with 7 signatures, including ADH1C, APOE, RAP1GAP, NPC1L1, P4HB, SOD2, and TNFSF10. This model is considered to be an independent prognosticator and is a more accurate predictor of overall survival than age, gender, and stage. TIDE score was increased in high-risk group, while the infiltration levels of CD8^+^ T cells and M1 macrophages were decreased. The number and intensity of intercellular communication were increased in the high ER stress group.

**Conclusions:**

We constructed and validated a novel prognostic model for pancreatic cancer, which can also be used as an instrumental variable to predict the prognosis and immune microenvironment. In addition, this study revealed the effect of ER stress on cell–cell communication in the tumor microenvironment.

**Supplementary Information:**

The online version contains supplementary material available at 10.1186/s12967-024-05158-y.

## Introduction

Pancreatic ductal adenocarcinoma (PDAC) is the most prevalent pancreatic malignancies, which is aggressively metastatic with one of the leading mortalities of cancer worldwide [1]. Majority of patients are diagnosed at an advanced stage with missed opportunity for surgery and poor prognosis [2]. The 5-year survival rate for PDAC patients is reported to be less than 10% [1]. Liver metastasis is the most common form of PDAC distant metastasis, which suggests poor prognosis for PDAC patients [3]. And liver metastasis can be influenced both by tumor cells and tumor microenvironment [4]. However, the further mechanisms underlying the regulation and promotion of PDAC cells metastasizing to liver tissues still remains to be elucidated.

Endoplasmic reticulum is an important place for regulating calcium homeostasis, lipid metabolism, protein synthesis, and post-translational modification [5]. Endoplasmic reticulum stress (ERS) is a condition in which many endogenous and exogenous damaging factors hinder the ability of ER to properly fold, secrete and modify post-translationally protein, resulting in an increased load of misfolded proteins in this organelle [6]. If endoplasmic reticulum stress keeps at a high level continuously, the responding cells engage in self-destruction. Nowadays, persistent and excessive ER stress is emerging as a key factor in many human diseases, including cancers [7]. Sustained ER stress can enhance tumor cell survival, angiogenesis, metastatic potential, drug resistance, and immunosuppression in tumors [8]. MYC-induced ER stress exhibit activation of unfolded protein response (UPR) in variety of human cancers, including lymphoma, neuroblastoma, prostate cancer, and breast cancer [9, 10]. Induction of ER stress and activation of UPR can inhibit the surface expression of major histocompatibility complex Class I (MHC-I) molecules thus affect the tumor immune microenvironment [11]. However, the role of endoplasmic reticulum stress in PDAC has been less reported, and the influence of endoplasmic reticulum stress in tumorigenesis and development, apart from the unfolded protein response, remains unclear.

Lipid metabolic disorders are one of the most salient metabolic alterations in cancer, which provide energy, biofilm components, and signal molecules required for proliferation, invasion, and metastasis of tumor cells [12]. It has been reported that inhibition of SCD1 leads to differentiation of liver tumor cells through ER stress-induced UPR [13]. Decreased Acetyl-CoA–producing enzymes (ACLY) expression reduces tumor cell viability and inhibits tumor progression in glioblastoma, melanoma cancer, PDAC and prostate cancer [14, 15]. Down-regulation of LDLR reduces cholesterol uptake and tumor proliferation in breast cancer cells, small cell lung cancer (SCLC) and PDAC cells [16–18]. Acyl-CoA oxidase 1 (ACOX1) enhances the response of HCC cells to oxidative stress through succinylation and increased activity [19]. Therefore, the disturbance of all aspects in lipid absorption, synthesis and hydrolysis is related to tumor occurrence and development. However, the role of lipid metabolism and endoplasmic reticulum stress in liver metastasis of PDAC needs to be further clarified.

The crosstalk between ER stress and lipid metabolism further exacerbates the malignant properties of pancreatic cancer [20]. ER stress can impact lipid metabolism by regulating the expression of key enzymes involved in lipid synthesis and metabolism [21]. Conversely, lipids can influence ER function and the UPR through various mechanisms, including the regulation of ER membrane composition and the modulation of ER stress response signaling pathways [22].

The heterogeneity of tumor microenvironment (TME) has great impact on prognosis [23]. TME plays an important role in the regulation of the immune response to cancer. Tumor cells and their microenvironment typically produce multitudinous immunomodulatory molecules that can negatively or positively affect immune cell function [24]. The recruited immune cells in TME can promote anti-tumor immunity and pro-tumor immunity, leading to tumor growth, escaping from immune surveillance and resistance to immunotherapy [25]. Hence, elucidating the alterations in the immune microenvironment during the liver metastasis of PDAC is crucial for enhancing patient prognosis.

This investigation identified ERS_Lipid-associated genes in PDAC and discerned liver metastasis-related genes using single-cell and bulk transcriptional analyses. Subsequently, a novel prognostic signature was devised and authenticated employing integrated machine learning algorithms. This risk signature demonstrated efficacy in immune trait categorization, tumor mutational burden (TMB) prognostication, and pharmaceutical selection. Furthermore, intercellular signaling was scrutinized in high and low ERS_Lipid cohorts to unveil the impact of ER stress and lipid metabolism on the tumor microenvironment and immune response.

## Method and materials

### Data collection

Original RNA_Seq data from TCGA_PAAD cohort was downloaded from TCGA database (https://portal.gdc.cancer.gov/). The transcriptome data of normal pancreatic cohort was downloaded from GTEx database (https://www.gtexportal.org). Transcriptome data from TCGA and GTEx were integrated and normalized between datasets to jointly screen for differentially expressed genes associated with pancreatic cancer. Gene expression microarrays of GSE71729, GSE34153, GSE28735, GSE57495, GSE62452 and GSE85916 were collected from GEO database (https://www.ncbi.nlm.nih.gov/geo/). Single cell transcriptional data from GSE154778 and GSE197177 were also obtained from GEO database. The gene expression data of PA_CA and PA_AU datasets were downloaded from ICGC database (https://dcc.icgc.org/). A total of 690 patients merged and batched from TCGA, ICGC, GEO (GSE28735, GSE57495, GSE62452 and GSE85916) with both clinical and gene expression information were randomly divided into a training set (*n* = 487) and a validation set (*n* = 203) through caret R package. Detailed information for the datasets mentioned above was shown in Additional file 2: Table S1.

### Identification of the endoplasmic reticulum stress and lipid metabolism related genes

The ssGSEA and consensus clustering methods were used to classify the endoplasmic reticulum stress and lipid metabolism related pathways and gene sets into 2 clusters. Then, WGCNA analysis was conducted to deeply screen out gene modules co-expressed with ER stress and lipid metabolism. Finally, the 521 lipid metabolic genes and 295 ER stress related genes from literatures and 495 genes from module purple were taken together, and 1240 genes were subjected to the following analysis.

### Identification of the liver metastasis related genes

Both single cell RNA data and bulk RNA data were used to identified the liver metastasis related genes. The ScRNA data was screened with nCount_RNA ≥ 1000, nFeature_RNA ≥ 200 & nFeature_RNA ≤ 10,000, percent.mt ≤ 20 and percent.rb ≤ 20. RNA_snn_res. = 1.5 was selected for downgrading and cell grouping. Both TSNE and UMAP methods were used to display the results of cell clustering. SingleR package (Version 2.4.1) was used to annotate cell types. WGCNA analyses (Version 1.72-5) were used to identify the co-expressed genes of liver metastasis in GSE71729 and GSE34153. Differentially expressed genes between primary tumor cells and liver metastatic cells were obtained by Seurat package (Version 5.0.3) in ScRNA dataset and by limma R package (Version 3.18) in bulk RNA dataset. Then, 1331 genes from both liver metastatic co-expressed genes and DEGs were identified. Ultimately, through the intersection of ER stress and lipid metabolism related genes, tumor related differentially expressed genes and liver metastasis-related genes, 92 potential prognostic genes were enrolled for subsequent analysis.

### Construction and validation of the novel prognostic signature

To develop a novel prognostic model to predict the overall survival of pancreatic cancer, an integration and combination of 10 machine learning algorithms [26] (CoxBoost, Lasso, stepwise Cox, plsRcox, Ridge, Enet, SurvivalSVMS, GBMs, SuperPC and RSF) were used to select the prognostic genes. Then, the gene coefficients were calculated by multivariate cox regression analysis. And the risk score of every patient was calculated following the formula: *Risk* *score* = *Σ* (*Coefi* × *Exp*). Next, all the patients in the train set were stratified into high- and low-risk group according to the optimal cut-off of risk score. Kaplan–Meier survival analysis was performed to compare the OS time between the high- and low-risk groups. The same calculating formula and cut-off were utilized in the test dataset and TCGA, ICGC cohorts. ROC curves, DCA curves and calibration curves were also drawn to evaluate the accuracy and consistency of the prognostic model by timeROC (Version 0.4), ggDCA (Version 1.1), survival (Version 3.5-8) and rms R (Version 6.8-0) packages.

### GSEA and KEGG enrichment analyses

The GSEA analysis was used to identify the enriched pathways in the high- and low-risk groups by clusterProfiler (Version 4.10.1) and enrichplot (Version 1.22.0) R packages. The top5 signal pathways were shown. The correlation of risk scores and prognostic genes with KEGG pathways of hallmarks in tumor were analyzed through GSVA analysis. The metabolism alterations of single cell transcriptomic data were conducted by scMetabolism [27] (Version wu-yc) R package (Additional file 5: Table S4).

### Tumor mutation burden and drug sensitivity analyses

The SNP information was collected from TCGA database. Then the gene mutation information in high- and low-risk groups was analyzed by maftools (Version 2.18.0) R package. The correlation between risk score and tumor mutation burden in TCGA cohort was also explored via survminer (Version 0.4.9) R package. And the drug sensitivity analysis between high- and low-risk groups was performed by pRRophetic [28] (Version 0.5) R package.

### Immune microenvironment analysis and cell–cell communication analysis

CIBERSORT algorithm [29] and ssGSEA method were utilized to provide a computational estimation for immune infiltration and tumor microenvironment. And immune functional pathways between two clusters were also analyzed via ssGSEA. TIDE [30] and TCIA [31] information of TCGA cohort in high- and low-risk groups were also analyzed by limma and ggpubr (Version 0.6.0) R packages. The cell–cell communication analysis of cells between high- and low-ERS groups were analyzed through CellChat (Version 1.6.1) R package [32].

### Cell culture

The cells used in this experiment were sourced from the laboratory of Department of General Surgery at Peking Union Medical College Hospital. HPNE and PANC1 cells were cultured in DMEM medium supplemented with 10% fetal bovine serum, while CAPAN1 cells were cultured in IMDM medium supplemented with 20% fetal bovine serum, the BXPC3 cell line was cultured in RPMI 1640 medium with 10% fetal bovine serum. The BXPC3 cell line was utilized as the parental cell strain to establish the first-generation liver metastasis cell line through a mouse model of liver metastasis induced by portal vein injection. A subset of the first-generation metastatic cell lines exhibited heightened tumor growth capabilities and significantly enhanced distant organ metastatic potential. Subsequently, we conducted repeated portal vein injections and screenings using these cell lines, extracted cells from liver metastatic foci post in vivo metastasis, further cultured and expanded them, leading to the derivation of the BXPC3_LMT cell line.

### Quantitative polymerase chain reaction (qPCR) and immunohistochemistry

RNA was extracted from five cell lines including HPNE, PANC1, CAPAN1, BXPC3 and BXPC3_LMT using TRIzol reagent (Takara, Japan). The extracted RNA (1000 ng) was reverse transcribed using the PrimeScript™RT kit (Takara, Japan). Real-time quantitative PCR was performed using SYBR Green Master Mix (Vazyme, China). Both the reverse transcription and q-PCR reaction systems were prepared according to the manufacturer’s instructions, and all procedures were conducted on ice. GAPDH was used as an internal control, and the primer sequences are listed in Additional file 3: Table S2. The relative gene expression levels were calculated using the 2^(− ΔΔCT) method.

Tumor tissues and corresponding adjacent tissues were obtained from 80 patients who received curative resection for PDAC at Peking Union Medical College Hospital in Beijing. Pathological verification of PDAC was achieved for all patients, and informed consent was acquired from all participants engaged in the research. The 4 μm paraffin sections were deparaffinized in xylene and rehydrated in graded alcohols. Antigen retrieval for SOD2, P4HB, and TNFSF10 utilized EDTA buffer (pH 9.0). Subsequently, the sections were treated overnight at 4 °C with SOD2 antibody (dilution 1:40,000, Proteintech, USA), P4HB antibody (dilution 1:200, Proteintech, USA), and TNFSF10 antibody (dilution 1:400, Proteintech, USA). DAB Plus reagent kit (Gene Tech, Shanghai, GK600705) was used for staining. The evaluation of all tissue sections was conducted using the ImageJ-based IHC Profiler plugin, categorizing the sections into three levels: positive (≥ 2+), low positive (1+), and negative (0). Additionally, the sections were independently assessed by two pathologists unaware of the sample identities, and all evaluations were manually corrected.

### Statistical analysis

The overall survival of patients in high- and low-risk groups was compared via the Kaplan–Meier analysis with the log-rank test. The identification of independent predictors for patient prognosis was performed through univariate and multivariate Cox regression analyses. Wilcoxon test was performed to ascertain the gene expression levels between two groups and assess the variance in TMB, drug sensitivity, immune scores, TIDE scores, and IPS scores between the two risk groups. And the correlation between the two groups was analyzed by spearman analysis. Each analysis was systematically repeated to ensure the reliability of the results. All statistical analyses were performed with R software (Version 4.3.0). All *p* values two-tailed with *p* < 0.05 in single tests and adjusted *p* < 0.05 in multiple tests were considered statistically significant.

## Results

### Identification of endoplasmic reticulum stress and lipid metabolism related genes in PDAC

**Fig. 1:**
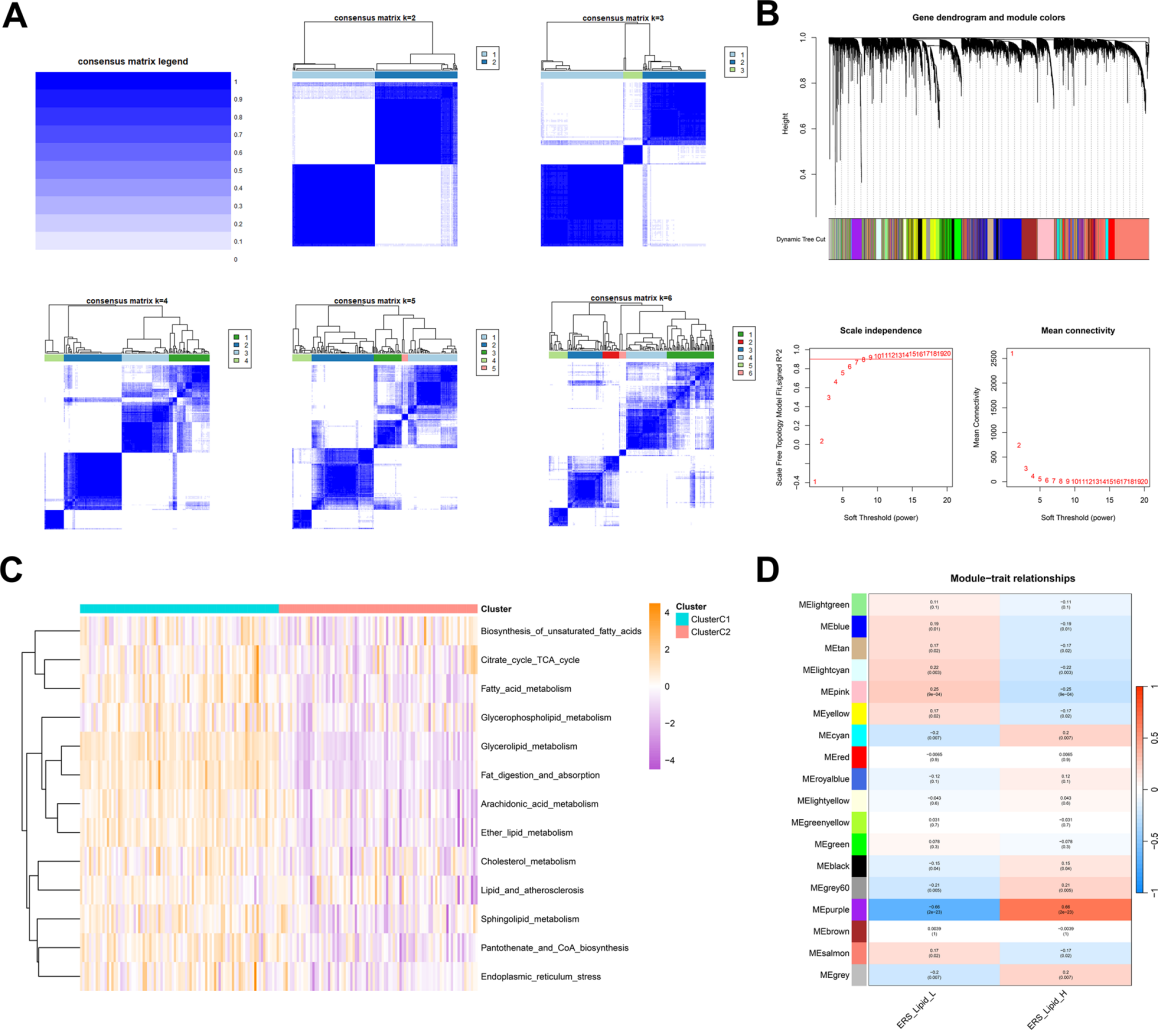
Identification of ER stress and lipid metabolism related genes. **A** Consensus clustering analysis of ER stress and lipid metabolic pathways, the TCGA data set is divided into two well-differentiated subgroups when *k* = 2. **B** This section describes related parameters of WGCNA analysis. **C** Heatmap and clustering of ssGSEA fractions of ER stress and lipid metabolism-related pathways. **D** WGCNA analysis screened out ERS_Lipid co-expressed gene modules

This study was conducted following the procedures shown in Additional file 1: Fig. S1. Persistent ER stress and lipid metabolic disturbance are novel features of malignancies. To investigate the prognostic significance of ER stress and lipid metabolism in pancreatic cancer, lipid metabolic signal pathways and ER stress gene set were analyzed by ssGSEA and consensus clustering (Fig. 1A, C). The consensus matrix heatmap showed that *k* = 2 was the optimal classification method, dividing PDAC samples into Cluster 1(sample size = 89) and Cluster 2(sample size = 89). The clustering heatmap in Fig. 1C showed that ER stress and lipid metabolism related genes and pathways had higher enrichment scores in Cluster1. WGCNA analysis was then performed to identify co-expressed genes involved in high and low ERS_Lipid subgroups in PDAC. In the process of co-expression network construction, we observed that the soft thresholding power β was 7 when the fit index of scale-free topology reached 0.9 and 18 modules were identified (Fig. 1B, D). The purple module was observed to have most significant correlations with ERS_Lipid in PDAC (Fig. 1D). Ultimately, 1240 ER stress and lipid metabolism related genes from both marker genes and WGCNA module genes were screened out for further analysis.

### Identification of liver metastasis related genes in PDAC

**Fig. 2:**
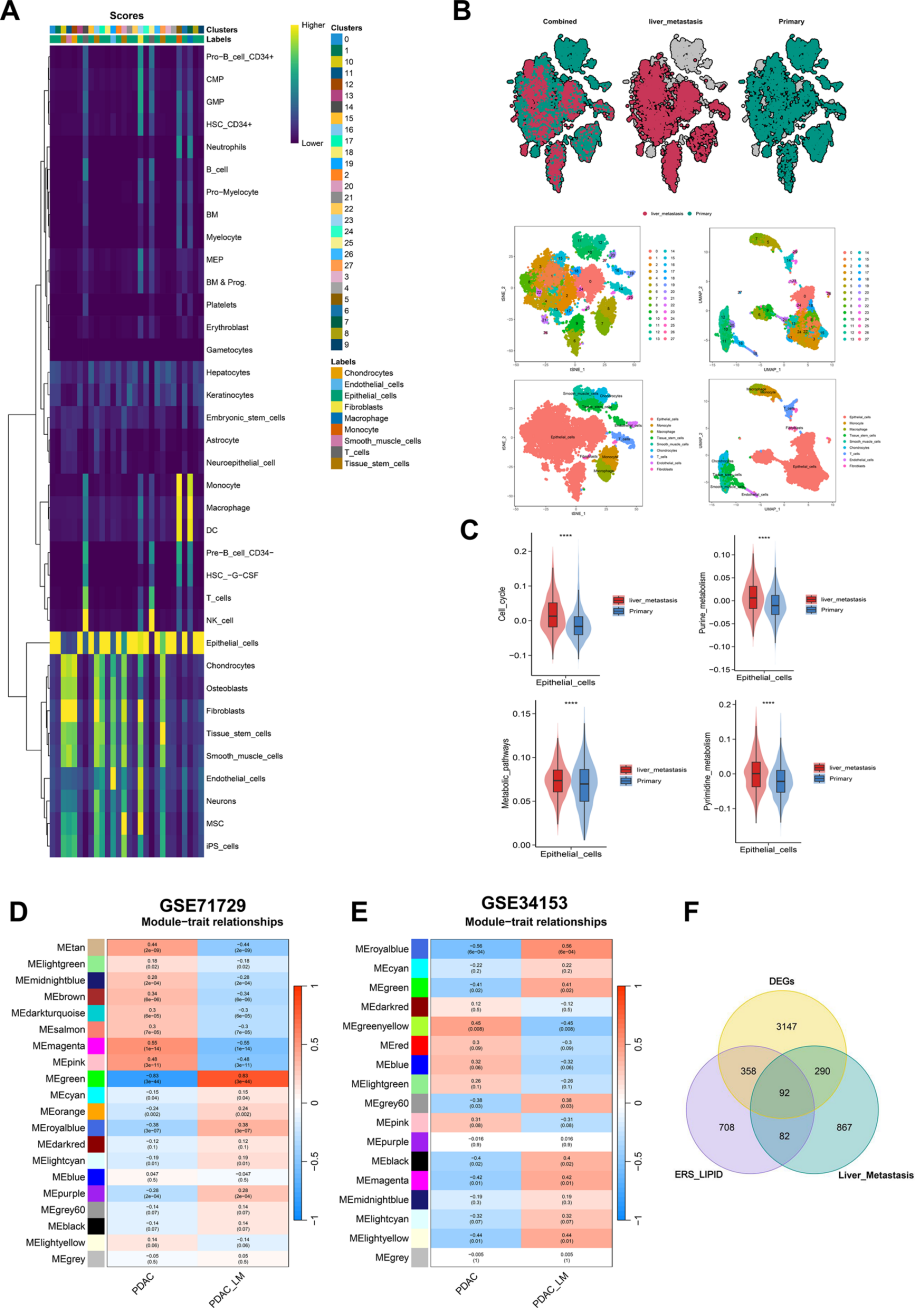
Identification of liver metastasis related genes. **A** Heatmap of cell scores. When the cell scores of a cluster significantly are higher in a reference cell type than other labels, they are annotated as that cell type. **B** Cell subpopulation clustering and annotation results of GSE154778 data set. **C** Alterations of cell cycle, purine metabolism, pyrimidine metabolism, and metabolic signaling pathways in epithelial cells between primary and liver metastasis groups. **D, E** WGCNA analysis screened the gene modules co-expressed with liver metastasis in GSE71729 and GSE34153. **F** The Venn diagram illustrated the presence of 92 shared genes

Liver metastasis is a devastating factor for the poor prognosis and high mortality of PDAC. In order to elucidate the specific mechanism and intrinsic driving factors of liver metastasis in PDAC, both single cell and bulk transcriptional analyses were conducted to select the differentially expressed genes between primary PDAC tissues and liver metastatic tissues (|log FC|> 1& FDR < 0.05; Fig. 2 and Additional file 1: Fig. S3). Each cell in the detecting samples is scored based on the cell type reference in SingleR package and the results of cell type were shown by the cell score heatmap (Fig. 2A). Nine cell types were identified in the single cell transcriptional analysis of GSE154778 cohort and 11 cell types were identified in GSE197177 cohort (Fig. 2B and Additional file 1: Fig. S3). The gene enrichment analysis showed that the scores of cell cycle, purine metabolism, pyrimidine metabolism and metabolic pathways were significant higher in liver metastatic epithelial cells than in primary epithelial cells (*p* < 0.0001, Fig. 2C). Estrogen response, androgen response, protein secretion and other signaling pathways were significantly upregulated in both primary and liver metastatic epithelial cells, while reversed in macrophages, monocytes and T cells. And the unfolded protein response was significantly enriched in liver metastatic epithelial cells, but not significantly changed in primary epithelial cells (Additional file 1: Fig. S3D, E). Liver metastatic co-expressed genes were also screened through WGCNA analysis in GSE71729 and GSE34153 cohorts (Fig. 2D, E). Finally, 722 liver metastatic DEGs from Single cell and bulk transcriptomic analyses, 857 liver metastatic co-expression genes from WGCNA analyses were identified. And 3887 DEGs between normal and tumor tissues in TCGA and GTEx cohorts were screened out through Limma R package with |log FC|> 1 and FDR < 0.05 (Additional file 1: Fig. S2). All the identified genes related to ERS_Lipid, liver metastasis and tumorous DEGs were intersected and 92 signatures were included in the subsequent analysis.

### Machine learning based integration constructs a prognostic model for PDAC

**Fig. 3:**
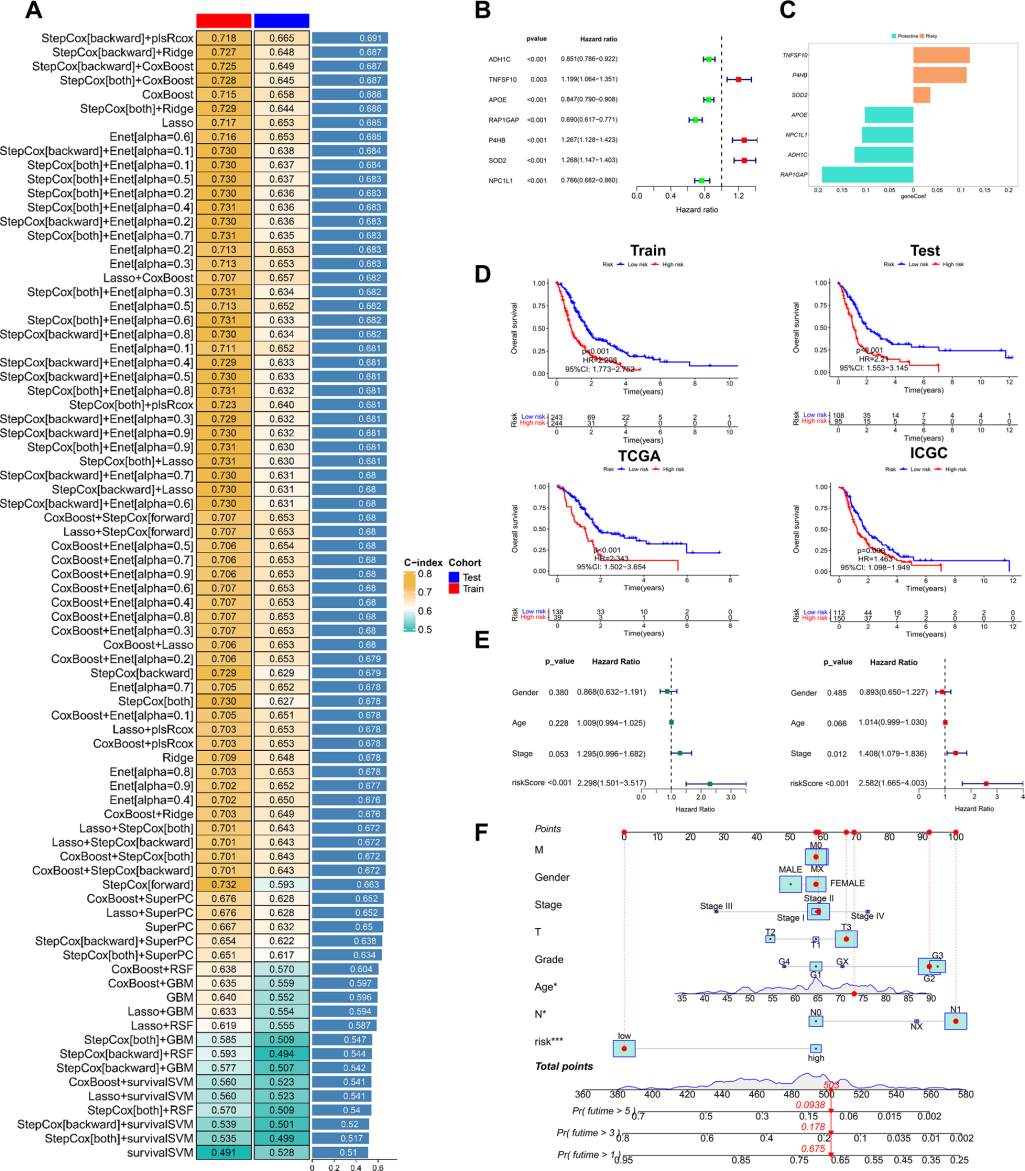
Construction and validation of a prognostic model for pancreatic cancer. **A** A combination of machine learning predictive models calculates the C-index for each model on the training set and the validation set. **B** Forest map of univariate cox regression analysis showed hazard ratios of the seven selected genes. **C** Coefficients of genes in the prognostic model. **D** Kaplan–Meier curves of OS in the train, test sets and TCGA, ICGC cohorts based on the model showed longer survival time in low-risk groups. **E** Univariate and multivariate Cox regression analyses were performed to assess the prognostic value of the risk score in conjunction with additional clinical features. **F** A nomogram combining risk scores and clinical information was constructed in the TCGA dataset

The 92 consensus genes screened above were subjected to the univariate Cox analysis and 42 prognostic genes were identified (Additional file 4: Table S3). Then the 42 genes were analyzed in a machine learning based integration program to establish a consensus ERS_Lipid signature for predicting the overall survival of PDAC patients. The leave-one-out cross-validation (LOOCV) framework was employed to fit a combination of 10 machine learning algorithms with hyperparameter tuning in the training set, and the C-index of each model was further calculated across the testing set (Fig. 3A). The optimal model was the combination of stepCox[backward] and plsRcox with the highest average C-index (0.691) in all model types (Fig. 3A). Seven consensus genes with leading prognostic value were identified and the gene coefficients were further calculated in the model (Fig. 3B, C). A risk score for each patient was then calculated with the expression of 7 genes weighted by their regression coefficients. According to the optimal threshold determined, all patients were divided into high-risk and low-risk groups. In order to further assess the influence of sample size on model accuracy, mitigate overfitting, and enhance generalizability and reliability, we also validated the model in the TCGA and ICGC datasets and found that the model functioned well in distinguishing the patients into high and low risk groups (Fig. 3D). Univariate cox regression analysis and multivariate cox regression analysis suggested that risk score can be used as an independent risk factor for the prognosis of PDAC patients (Fig. 3E and Additional file 1: Fig. S4). A nomogram was also established to take both clinical variates and risk score into account for clinical use (Fig. 3F). Stratifying PDAC patients based on clinical characteristics, the study validated the prognostic predictive ability of the ERS_Lipid signature across different clinical subgroups. The results demonstrated that the ERS_Lipid signature exhibited good prognostic discrimination in patients of varying ages, genders, grade stages, and T, N, M classifications (Additional file 1: Fig. S4). These findings suggested the prognostic model exhibited robustness and generalizability across diverse patient populations.

### Validation and evaluation of the novel model and the related gene enrichment analyses

**Fig. 4:**
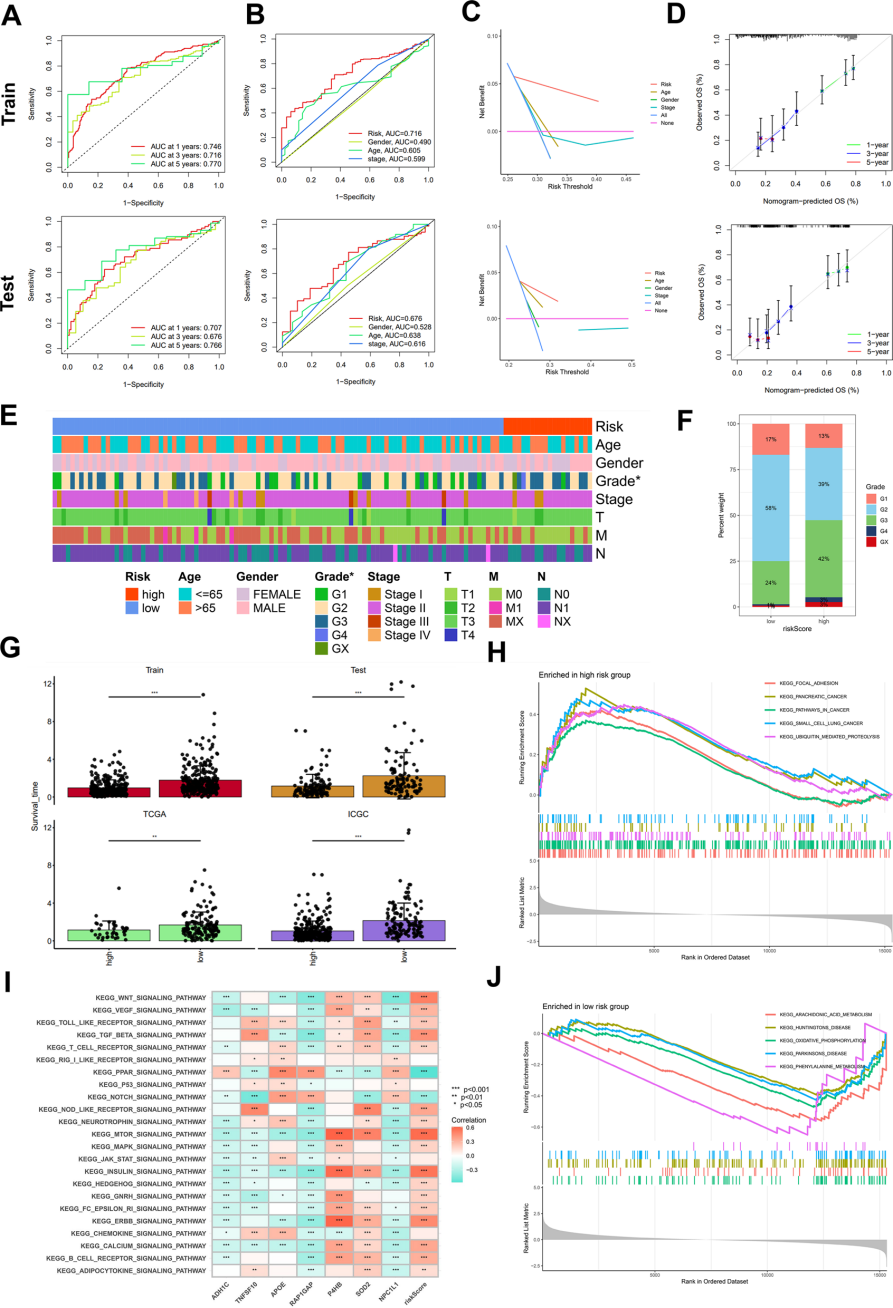
Evaluation of the novel prognostic model. **A** The ROC curves showed the 1-year, 3-year, and 5-year survival prediction accuracy of the model in the training and testing sets. **B** AUC value of risk score was higher than that of clinical characteristics. **C** The DCA curves showed the respective benefit of risk score and other clinical features in predicting clinical outcomes. **D** The 1-, 3-, and 5-year calibration curves assessed the predictive robustness of the model. **E, F** Risk score was significantly associated with the Grade classification of patients in the TCGA cohort. **G** Survival time in the high- and low-risk groups. **H, J** The Top5 signal pathways enriched in high- and low-risk groups with adjusted *P* value < 0.05. **I** GSVA analysis showed relationship between KEGG pathways and the model genes

To further evaluate the prognostic value of the established model, receiver operating characteristic (ROC) curves were plotted and the area under the ROC curve (AUC) was calculated at 1, 3 and 5 years, respectively. The results suggested that the prognostic accuracy of risk score was significantly higher than that of age, gender and stage (Fig. 4A, B). The Decision Curve Analysis (DCA) indicated that the clinical benefit of risk score in evaluating prognosis of PDAC was greater than that of age, gender and stage (Fig. 4C). The calibration curves of risk score at 1-, 3- and 5-year showed that risk score was robust in predicting the survival time in the training and testing sets (Fig. 4D). The risk scores derived from this predictive model exhibited a significant positive correlation with tumor grade, with a notably higher proportion of G3-4 patients observed in the high-risk group compared to the low-risk group (Fig. 4E, F). These findings suggest an association between this risk features and the clinical progression of PDAC patients. Patients in high-risk group had shorter survival time than in low-risk group across all the training and validation cohorts (Fig. 4G). The GSEA analysis was also performed to elucidate the signal pathways alteration in high- and low-risk groups. The results showed that focal adhesion, pancreatic cancer, pathways in cancer and ubiquitin mediated proteolysis were enriched in high-risk group, while arachidonic acid metabolism, oxidative phosphorylation and phenylalanine metabolism were enriched in patients with low-risk scores (Fig. 4H, J and Additional file 5: Table S4). And the correlation between risk scores and hallmark signal pathways was analyzed with GSVA algorithm. Risk score was significantly positively correlated with most of the tumor-related signaling pathways, such as MTOR, Insulin, ERBB and Wnt signal pathways, while significantly negatively related to PPAR and Notch signaling pathways (Fig. 4I and Additional file 1: Fig. S5).

**Fig. 5:**
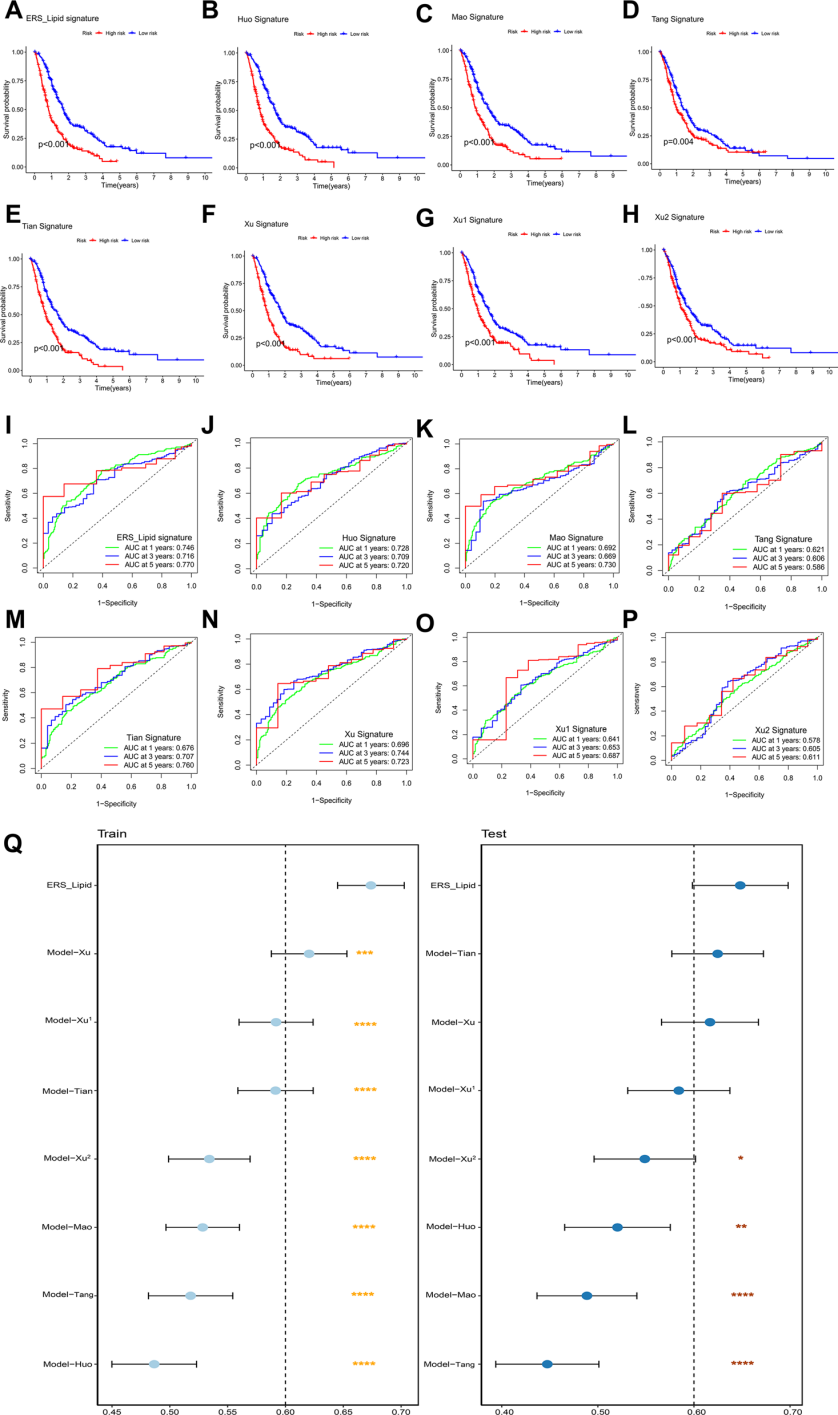
Comparison of the ERS_Lipid-related prognostic model with seven published prognostic models. **A–H** The Kaplan–Meier survival curves demonstrated that both the ERS_Lipid signature and the seven previously published prognostic signatures exhibit significant prognostic discrimination in the integrated training dataset. **I–P** The area under the ROC curve of the ERS_Lipid signature was greater than that of the other seven previously published signatures. **Q** ERS_Lipid signature demonstrated a higher C-index value in both the integrated training and validation datasets. “*”*p* < 0.05, “**”*p* < 0.01, “***”*p* < 0.001, “****”*p* < 0.0001

Furthermore, the comparison of the constructed model with 7 previously published prognostic models were also conducted [33–39]. All signatures exhibited excellent prognostic discrimination, with significantly lower survival times observed in the high-risk group compared to the low-risk group (Fig. 5A–H). However, the ERS-Lipid signature demonstrated larger AUC values at 1, 3, and 5 years compared to the seven previously published signatures, indicating higher prognostic accuracy in PDAC (Fig. 5I–P). Additionally, the ERS-Lipid signature displayed higher C-index values in both the entire training cohort and validation cohort, suggesting its superior robustness (Fig. 5Q). The results suggested that the ERS_Lipid signature functioned well in predicting the survival outcomes of patients with PDAC (Figs. 4, 5).

### Analyses of tumor mutation burden and drug sensitivity between high- and low-risk groups

**Fig. 6:**
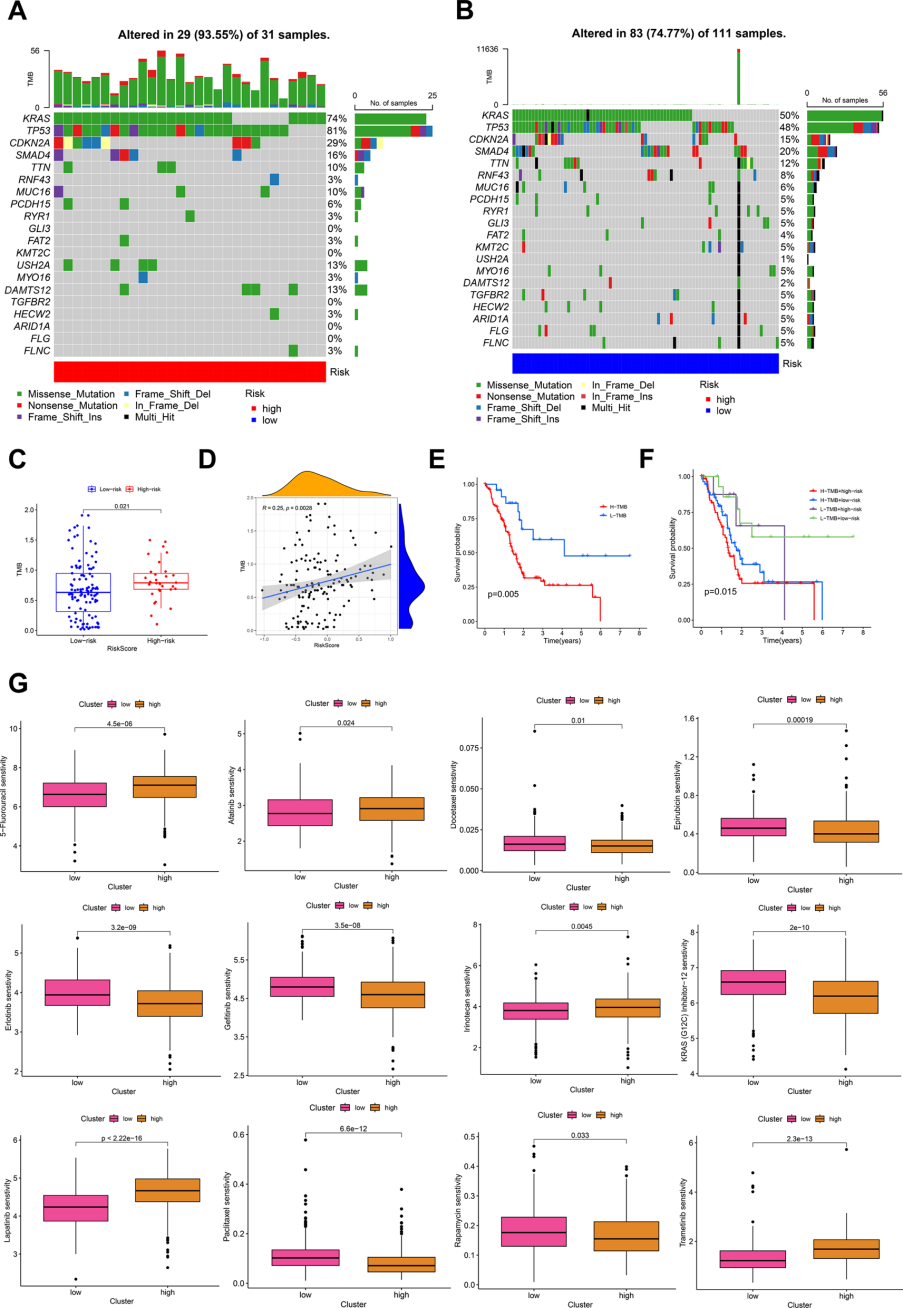
Analyses of tumor mutation burden and drug sensitivity in high-low risk groups. **A, B** The accumulated alteration of mutated genes in high- and low-risk groups. **C, D** TMB was positively related to risk scores. **E, F** Kaplan_Meier curves showed TMB and risk scores correlated with adverse prognosis. **G** The drug sensitivity in high- and low-risk groups. The *Y*-axis showed the IC50 value, which was negatively correlated with drug sensitivity

In order to further explore the correlation between tumor mutation burden (TMB) and risk score, the somatic mutation landscape of each PDAC sample in the high- and the low-risk groups were visualized by waterfall diagram, and it was observed that the top 20 mutant genes in the two subgroups were basically the same, but the total mutation load was 93.55% in the high-risk group and 74.77% in the low-risk group (Fig. 6A, B). We found the top 4 genes KRAS, TP53, CDKN2A, and SMAD4 with the highest mutation load in the high- and low-risk groups. For the classification of variation, missense mutation, nonsense mutation and frame shift del are the top 3 across all mutation types. The results also showed that risk score had significantly positive correlation with TMB (Fig. 6C, D). And the survival time of patients in high-risk score and high TMB group was shorter than that of patients with low-risk score and low TMB (Fig. 6E, F). The drug sensitivity analysis in the high- and low-risk groups showed that the risk score was correlated with multiple drug sensitivity (Fig. 6G). The results showed that the sensitivity to 5-Fluorouracil, Afatinib, Irinotecan, Lapatinib and Trametinib in patients with high-risk scores was lower than in low-risk group, while reversed results were observed in the sensitivity to some drugs, such as Docetaxel, Epirubicin and Gefitinib (Fig. 6G).

### Analysis of tumor immune landscape between high- and low-risk groups

**Fig. 7:**
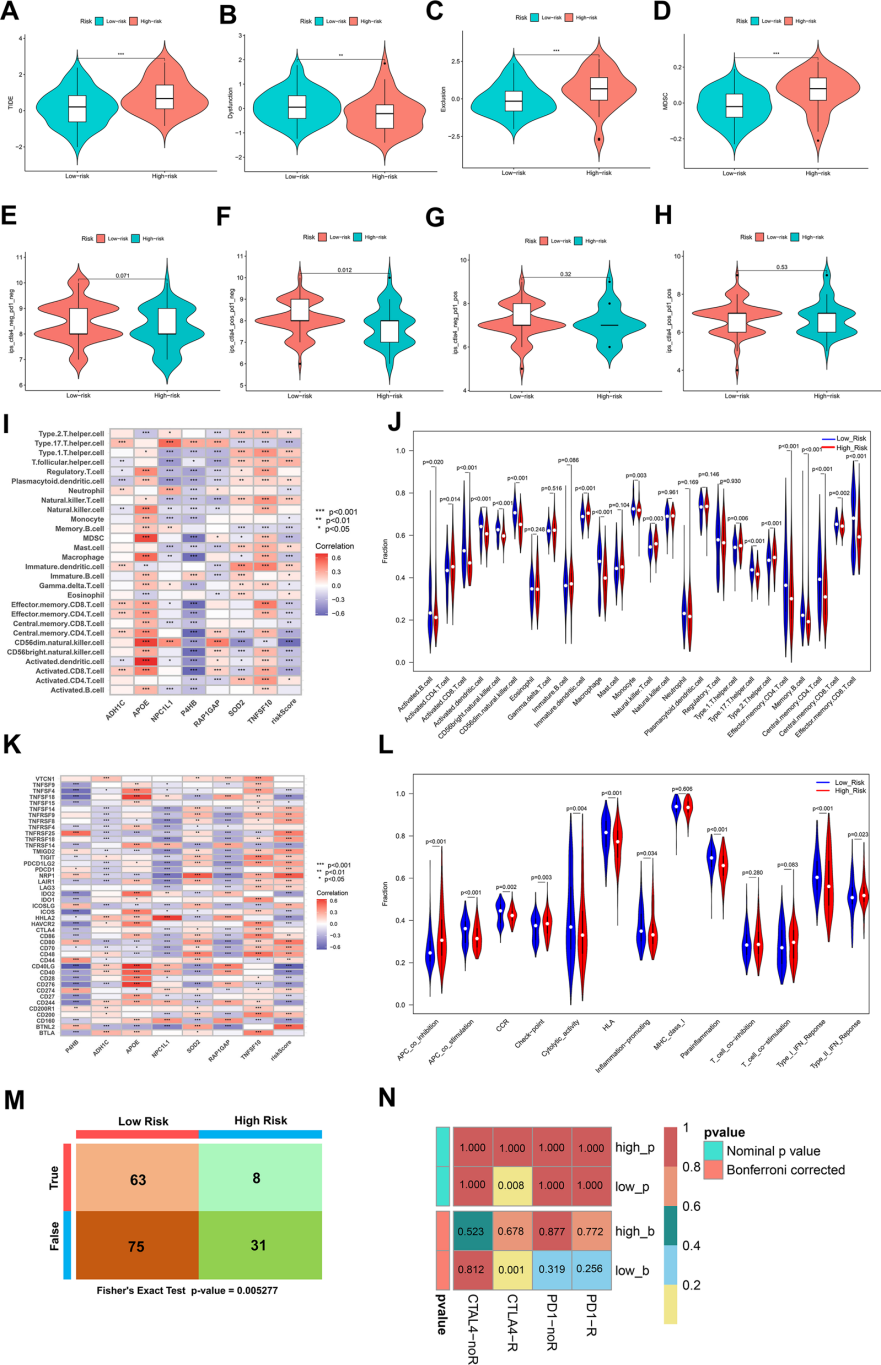
Immune landscape in high- and low-risk groups. Alteration of **A** TIDE score, **B** immune dysfunction, **C** immune exclusion and **D** MDSC in high- and low-risk groups. **E–H** IPS scores of PD1 and CTLA4 in high- and low-risk groups. **I, J** The immune infiltration variations in high- and low-risk groups. **K** Risk scores correlated with immune checkpoints. **L** Immune function altered in high- and low-risk groups. **M** Fisher’s test revealed the cohort distribution of immunotherapy response among patients categorized into high and low-risk groups within the TCGA dataset. **N** The responsiveness of patients to immune checkpoint inhibitor (ICI) treatment based on submap algorithm and the TIDE scores with Bonferroni corrected *p* values

The heterogeneity of tumor immune microenvironment is an important factor affecting tumor development and prognosis. To assess the immune landscape in high- and low-risk groups, we analyzed the alteration of TIDE [30] scores and IPS scores (Fig. 6A–H). The results showed that TIDE score, immune exclusion score and MDSC score are higher in the high-risk group than in low-risk group, indicating higher likelihood of immune escape (Fig. 7A–D). And IPS of CTLA(+)PD1(−) was significantly lower in high risk group, while IPS of CTLA(+)PD1(+), CTLA(−)PD1(+) and CTLA(−)PD1(−) had no significant difference in high- and low-risk groups (Fig. 7E–H). This suggested that patients in the high-risk group may not respond well to immunotherapy. The immune infiltration alteration was also explored through CIBERSORT and ssGSEA analyses, which offered a computational estimation within the tumor microenvironment. The results showed that the infiltration levels of CD8^+^ T cells, M1 macrophages, T follicular helper cells, T regulatory cells and resting Dendritic cells were down-regulated in high-risk group, while that of plasma cells, resting CD4^+^ T cells memory, activated CD4^+^ T cells memory, resting NK cells and activated Dendritic cells were reversed (Additional file 1: Fig. S6). The enriched scores of immune infiltration and immune function showed that immune cells such as activated B cells, activated CD8^+^ T cells, CD56 bright NK cells, CD4^+^ central memory T cells and CD8^+^ effector T cells were significantly down-regulated in high-risk group. And The enrichment scores of activated CD4^+^ T cells, natural killer T cells, Th1 helper cells and Th2 helper cells was higher in the high-risk group (Fig. 7I, J). The correlation of check-point genes with risk score and prognostic genes was also analyzed and displayed in Fig. 7K. As for the immune functions, APC_co_inhibition, check point and type II IFN response signal pathways were upregulated in high-risk group, while immune pathways such as APC_co_stimulation, CCR, cytolytic activity and type I IFN response signal pathway were decreased in high-risk group (Fig. 7L). Furthermore, Fisher’s test revealed significant differences in the cohort distribution of response to immunotherapy among patients in high- and low-risk groups (Fig. 7M). Submap analysis provided additional validation that patients classified in the low-risk group exhibited a higher responsiveness to immunotherapy targeting CTLA4 compared to those in the high-risk group (adjusted *p* value < 0.05; Fig. 7N) (Additional file 6: Table S5).

### Cell–cell communication analysis between high- and low-ERS groups

**Fig. 8:**
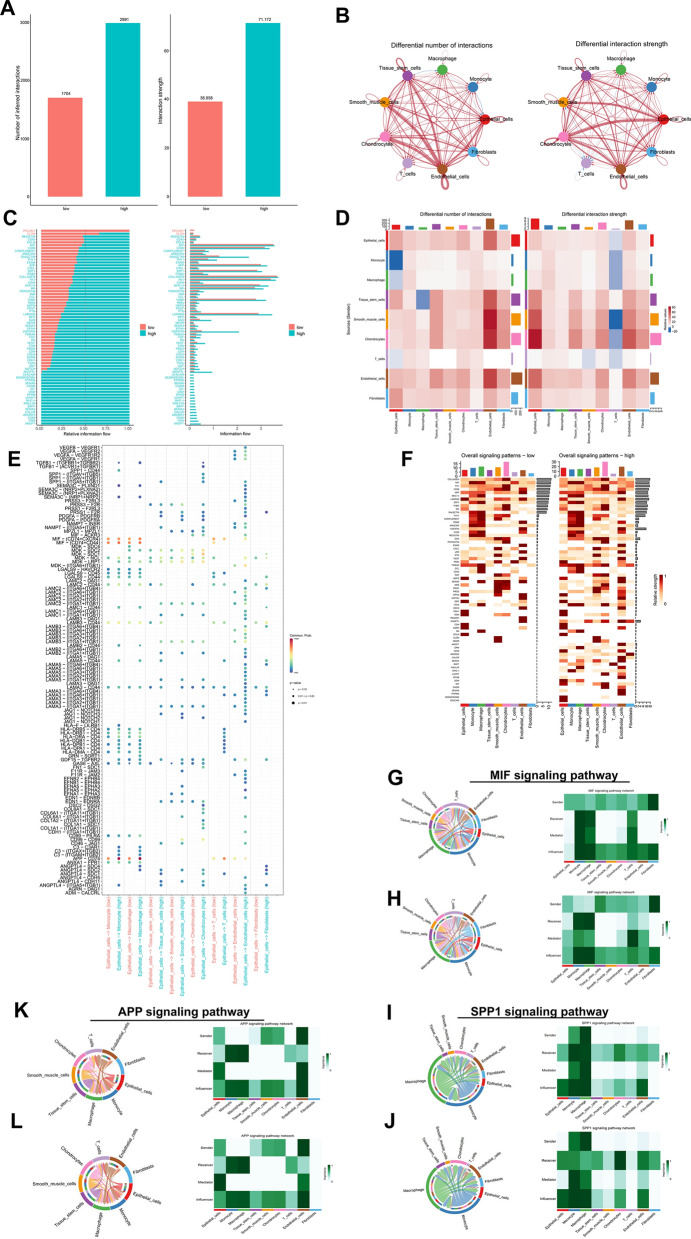
Cell–cell communication analysis between high- and low-ERS groups. **A** The number and strength of cell interaction mediated by individual signal pathways in high and low ERS groups. **B** Circle plots of communicating number and strength between immune cells and tumor cells. **C** The ranking bar chart showed the signal axes of interactive networks in high- and low-ERS groups. The signaling pathway with red labels was more abundant in the low-ERS group, the signaling pathway marked black was equally abundant in both groups, and the signaling pathway with green labels was more enriched in the high-ERS group. **D** Heatmap of cell–cell communication number and strength. Blue indicated reduced intercellular communication in the high-ERS group compared to the control group, while red indicated enhanced intercellular communication. **E** Bubble map of altered cell–cell communication mediated by individual signaling axes, with the horizontal axis showing the cell class that initiates and receives the signal, and the vertical axis showing receptor-ligand pairs of the signaling pathway. **F** Heatmaps displayed the overall (both outgoing and incoming) signal flows of each cell population. **G, H** MIF signal pathway, **I, J** SPP1 signal pathway and **K, L** APP signal pathway in low- and high-ERS groups

CellChat algorithm [32] was applied to explore and estimate the intercellular signaling communications based on gene expression information from single-cell transcriptomics. Communication patterns between high- and low-ERS groups were compared to predict pathological changes in cell–cell communication of PDAC. The results showed that the number and interaction strength of cell–cell communication were increased in high- ERS group (Fig. 8A, B). The interaction number and strength of epithelial cells (sender) to macrophages and monocytes were augmented, while the interaction number of monocytes, macrophages and T cells (sender) to epithelial cells and the interaction strength of other cells such as epithelial cells (sender) to T cells were down-regulated in high-ERS group (Fig. 8D). The information flows of each signaling pathway were then calculated to identify the communicating probability over all the pairs of cell types and the information flow between epithelial cells and other cells were further quantified (Fig. 8C, E). CLDN pathway was decreased, whereas some others like RESITIN, MIF, and SPP1 were increased in high-ERS group. And PECAM1 signal pathway was turned off, while some pathways, such as CEACAM, DESMOSOME and PTPRM signal pathways were turned on in high-ERS group (Fig. 8F, G). We selected three interesting signaling pathways for further analysis. The communicating interactions in MIF, APP and SPP1 signaling pathways among all cell types in the high- and low-ERS groups were analyzed and shown in Fig. 8G–L. We also investigated alterations of cell–cell communication in the high-low lipid metabolism groups. The number and intensity of cell–cell interactions in the high-lipid metabolism group also increased significantly. Concurrently, some signaling pathways related to tumor proliferation and progression, such as NOTCH, APP, and CEACAM, were also significantly activated in the high-lipid metabolism group (Additional file 1: Fig. S7).

### Metabolism reprogramming analysis based on the single cell transcriptomes

**Fig. 9:**
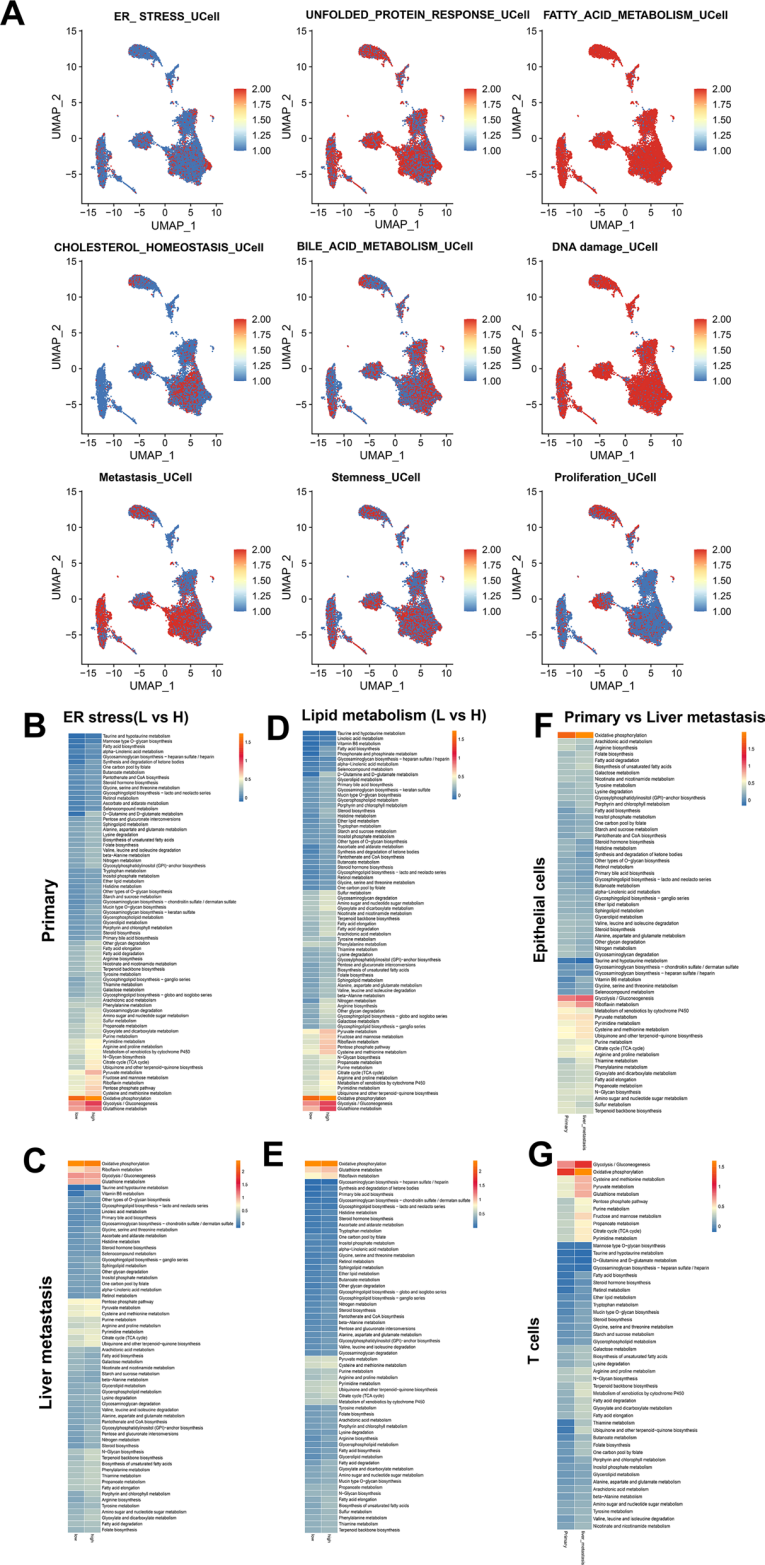
Metabolic alterations analyses based on single cell RNA_seq data. **A** Enrichment strength of signaling pathways related to endoplasmic reticulum stress, lipid metabolism, and malignant behavior in the pancreatic cancer single-cell dataset as depicted in the feature plot. **B, C** Metabolism reprogramming pathways in the high- and low-ER stress groups in primary and liver metastatic cells. **D, E** Metabolism reprogramming pathways in the high- and low-lipid metabolism groups in primary and liver metastatic cells. **F** The metabolic changes of epithelial cells in primary and liver metastatic groups. **G** The metabolic alterations of T cells in primary and liver metastatic groups

Analysis of the PDAC single-cell dataset using the UCell (Version 2.6.2) R package revealed active ER stress signals across all cellular subtypes, particularly prominent in Epithelial cells. Furthermore, downstream adaptive pathways of endoplasmic reticulum stress, such as the unfolded protein response, exhibited significant activation in all cellular subtypes. Concurrently, lipid metabolism-related pathways, including fatty acid metabolism, cholesterol homeostasis, and bile acid metabolism, were notably enriched in various cellular subtypes, with a pronounced enrichment observed in Epithelial cells. Notably, there was no discernible difference in DNA damage among the different PDAC cellular subtypes. Malignancy-associated gene sets related to metastasis, stemness, and proliferation demonstrated higher enrichment scores in Epithelial cells, tissue stem cells, other stromal cells, and macrophages (Fig. 9A). To further elucidate the metabolic alterations between high- and low-groups of ER stress and lipid metabolism, we investigated the metabolic pathways by scMetabolism R package [27]. Apart from phenylalanine metabolism, taurine and hypotaurine metabolism, the majority of metabolic pathways from primary tumor cells were activated in the high-groups of both ERS and lipid metabolism, such as purine metabolism, pyrimidine metabolism, steroid biosynthesis, nitrogen metabolism and fatty acid biosynthesis (Fig. 9B, D). Most metabolic pathways in liver metastatic cells were also significantly up-regulated in the high-ERS and high-lipid metabolic groups, except oxidative phosphorylation, glycosphingolipid biosynthesis, and one carbon pool by folate (Fig. 9C, E). The metabolic changes of tumor cells represented by epithelial cells and immune cells represented by T cells in the primary and liver metastasis groups were also significant (Fig. 9F, G).

### Validation of the expression and prognostic value of ERS_Lipid-related hub genes

**Fig. 10:**
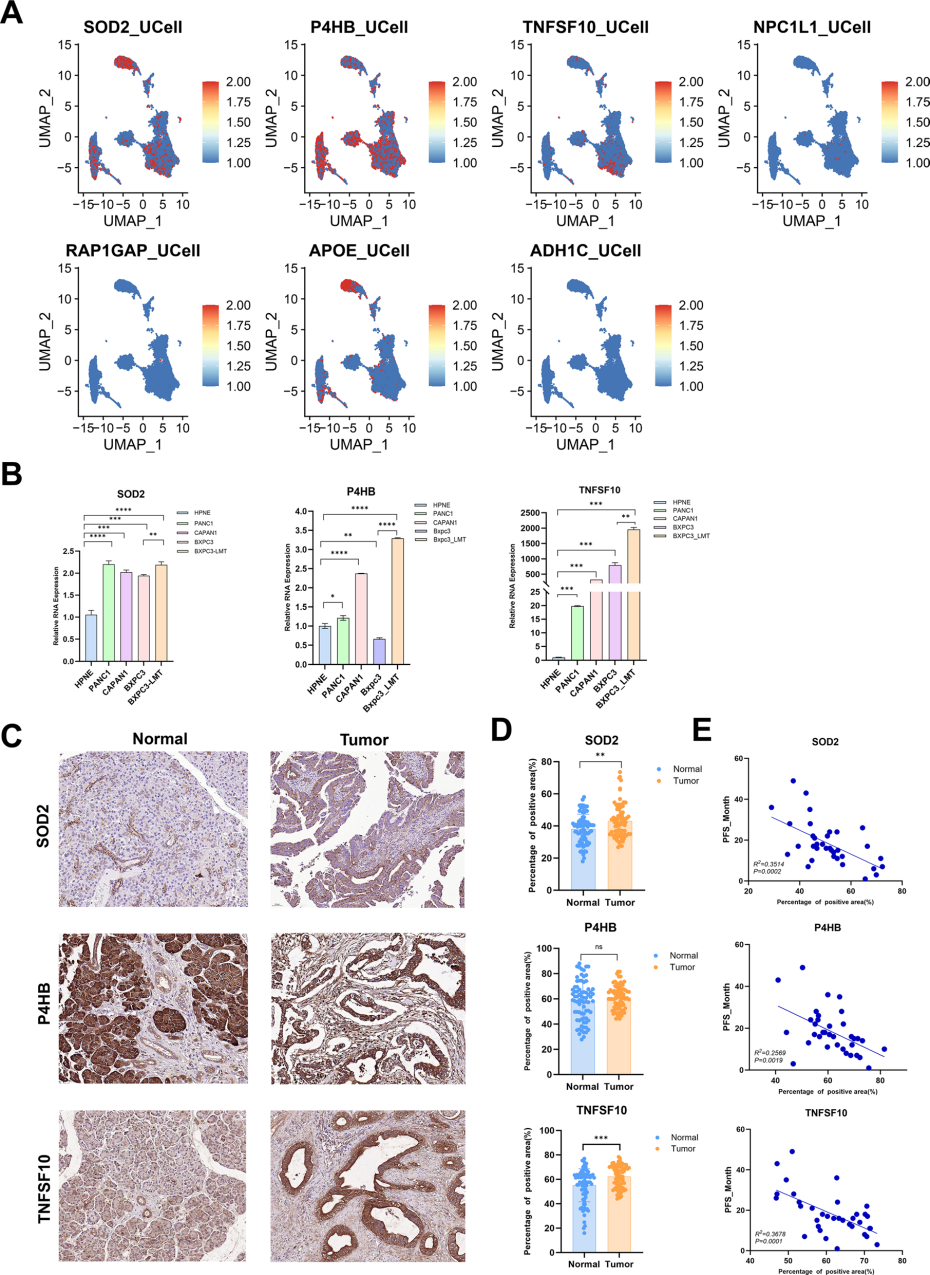
Validation of the expression levels and prognostic relevance of hub genes. **A** Gene expression levels of ERS_Lipid signature in single-cell dataset. **B** Relative RNA expression of SOD2, P4HB and TNFSF10. **C, D** The protein expression levels of SOD2, P4HB and TNFSF10 were detected by immunohistochemical staining in 80 pairs of PDAC tumor tissues and non-tumor tissues using multiple tests with adjusted *p* values. **E** Correlation analysis of the hub gene expression levels with prognosis in PDAC. “ns” adjusted *p* value > 0.05, “*” adjusted *p* value < 0.05, “**” adjusted *p* value < 0.01, “***” adjusted *p* value < 0.001, “****” adjusted *p* value < 0.0001

In this investigation, a prognostically significant ERS_Lipid signature comprising seven genes was identified through a combination of machine learning algorithms. Notably, among these genes, SOD2, P4HB, and TNFSF10 were identified as hub genes associated with adverse prognostic outcomes in pancreatic cancer. To further validate the predictive value of the ERS_Lipid signature, experimental validation was conducted to assess the expression levels and prognostic relevance of the three risk genes. Single-cell sequencing data indicated widespread expression of SOD2 and P4HB across various cell populations, with TNFSF10 primarily expressed in epithelial cells, and APOE predominantly expressed in macrophages and a small subset of stromal cells. NPC1L1, RAP1GAP, and ADH1C show relatively low expression levels across different cell populations (Fig. 10A). The qPCR results showed that the expression of SOD2 and TNFSF10 was elevated in the PANC1, CAPAN1, BXPC3, and BXPC3-LMT cell lines compared to HPNE. The expression of P4HB was increased in PANC1 and CAPAN1, decreased in BXPC3, while significantly upregulated in BXPC3-LMT (Fig. 10B). The protein expression of the 3 hub genes was examined by IHC to determine significant differences between the PDAC group (*n* = 80) and the normal group (*n* = 80). SOD2 exhibited a total positive rate of 75% (60/80) in the PDAC group and 63.75% (51/80) in the normal group. P4HB showed a total positive rate of 96.25% (77/80) in the PDAC group and 100% (80/80) in the normal group. TNFSF10 displayed a total positive rate of 95% (76/80) in the normal group and 100% (80/80) in the PDAC group (Fig. 10C). The positive areas of SOD2 and TNFSF10 in the PDAC group were significantly higher than those in the normal group, while the positive areas of P4HB showed no significant change (Fig. 10D). However, P4HB is predominantly expressed in acinar cells in normal tissues, with significantly lower expression levels observed in pancreatic ductal cells compared to tumor tissues. Furthermore, the correlation between the positive area of each protein in tissues and progress free survival (PFS) time was examined in 35 patients with complete follow-up data. The findings revealed a negative correlation between SDO2, P4HB and TNFSF10 expression levels and prognosis in PDAC patients. (adjusted *p* value < 0.05, Fig. 10E).

## Discussion

Endoplasmic reticulum homeostasis plays a pivotal role in regulating cell function and viability. The ER homeostasis of malignant cells, stromal cells as well as infiltrating immune cells experience perturbations by carcinogenic, transcriptional and metabolic abnormalities and other stimuli, which collectively contribute to an adverse microenvironment in response to sustained ER stress [40]. ER stress is mainly manifested by accumulation of misfolded and unfolded proteins, which can promote antioxidant reaction through ATF4 and NRF2, reducing oxidative stress and promoting tumor metastasis [41]. Some studies have shown that ER stress in cancer cells can regulate the functions of T cells, dendritic cells, natural killer (NK) cells, etc., coordinate various immune escape mechanisms, and mediate tumor growth and metastasis [42–44]. Lipid anabolism and catabolism as well as lipid distribution are regulated by ER to some extent. Both lipid metabolic reprogramming and ER stress can interact with other cellular functions and promote the development of diseases [45, 46]. Further study of ER stress and lipid metabolism may contribute to new understanding of tumor pathogenesis.

Pancreatic cancer is highly metastatic and refractory to the existing treatment including the targeted and immune therapy [47]. Tumor metastasis is a complex process driven by the combination of intrinsic properties of tumor cells (such as mutation burden) and crosstalk between tumor cells and other immune cells, stromal cells, and extracellular matrix in tumor microenvironment [48]. PDAC is characterized by a complex tumor microenvironment and is one of the least immune infiltrated cancers [49]. Liver is not only the main site of distant metastasis of PDAC cases, but also the main site of metabolism. Metabolic reprogramming and the heterogeneity of the tumor immune microenvironment both play important roles in tumor development and metastasis to the liver [50]. At present, the molecular mechanisms driving liver metastasis of PDAC have not been fully clarified, and further research is needed to contribute for the development of corresponding therapies and improving the prognosis of PDAC patients.

In this study, we screened out the ER stress and lipid metabolism related genes as well as the liver metastatic genes and developed a novel prognostic model for PDAC patients with seven genes based on bioinformatics and machine learning methods, which may provide a potential biomarker for the diagnosis and prognosis of PDAC patients with liver metastasis. The results of single cell transcriptome analysis showed that the pathways of cell cycle, purine metabolism, pyrimidine metabolism and metabolic pathways were upregulated in liver metastatic epithelial cells than in primary epithelial cells, which suggested the malignant progression in the liver metastasis of PDAC. The prognostic model was validated in the test set and the TCGA, ICGC cohorts. The results suggested that the risk score functioned well than the clinical features, such as age, gender and stage, in predicting the survival time of patients. The univariate and multivariate cox regression analyses also demonstrated that risk score can be used as an independent prognosticator in PDAC. Comparing with seven previously published models, including a broad metabolic signature, a hypoxia signature, an immune-related signature, a cuproptosis signature, a ferroptosis signature, a cholesterol metabolism-related signature, and a multi-omics signature covering various research directions, we found that the novel ERS_Lipid signature demonstrates significant advantages in prognostic accuracy and robustness for PDAC. This highlights the superiority of machine learning integrated screening methods and underscores the substantial role, as well as prognostic potential of ERS_Lipid in the occurrence and development of PDAC. Further gene enrichment analyses between the high- and low-risk subgroups also showed that pathways in cancers were increased in the high-risk group, and the hallmarks of tumor significantly correlated with risk scores. Patients in high-risk group are prone to have greater TMB, which provided evidence for the accuracy of this prognostic model and indicated that patients with high-risk scores are characterized by higher genetic heterogeneity. And the drug sensitivity analysis found that the sensitivity of patients in the high- and low-risk group to different drugs varied significantly, which can provide a certain guidance for the drug selection of adjuvant chemotherapy and targeted therapy for PDAC patients who lost the opportunity of surgery and after PDAC surgical resection.

The selected seven prognostic genes were APOE, ADH1C, RAP1GAP, NPC1L1, TNFSF10, SOD2 and P4HB, in which the gene set of APOE, ADH1C, RAP1GAP and NPC1L1 served as protective factors and gene set of TNFSF10, SOD2 and P4HB played as risky factors in the prognosis of PDAC. The differentially expression levels and the prognostic value of these model genes were also significant (Fig. 10 and Additional file 1: Fig. S8). Apolipoprotein E (APOE) is a secreted protein involved in lipoprotein metabolism and cholesterol transport. It has been demonstrated that APOE plays a role in regulating the abundance of MDSC and anti-tumor immunity [51]. Pencheva [52] et al. also reported that APOE inhibits invasion and angiogenesis in melanoma by attracting tumor cell LRP1 receptors and endothelial cell LRP8 receptors, respectively. Alcohol dehydrogenase 1C (ADH1C) is a member of the alcohol dehydrogenase family that metabolizes ethanol, fatty alcohols, and lipid peroxidation products [53]. ADH1C is associated with a poor prognosis for liver cancer and lung adenocarcinoma [54, 55]. In colorectal cancer, ADH1C acts as a tumor suppressor gene [53]. Many results of studies on the association of ADH1C with multiple tumors are significant but inconsistent. The role of ADH1C in pancreatic cancer is rarely reported and needs further study. Tumor suppressor gene RAP1GAP, which is inactivated by hypermethylation of its regulatory region, can cause thyroid tumor [56]. The silencing of RAP1GAP promotes endometrioid adenocarcinoma cell migration and invasion [57]. In addition, a research by Agarwal [58] et al. on non-alcoholic fatty liver disease suggested that RAP1GAP can reduce the expression of genes involved in fat synthesis in obese mice, indicating a correlation between RAP1GAP and lipid metabolism. Niemann-Pick C1-like 1 (NPC1L1) is a cholesterol transporter that plays a crucial role in intestinal absorption of cholesterol [59]. And NPC1L1 promotes the absorption of vitamin E and can interact with lipid peroxidation free radicals to prevent oxidative stress [60]. TNFSF10 has an inhibitory function in regulating breast cancer cell metastasis, but it has been reported that TNFSF10 can enhance the invasion of PDAC cells in vitro and increase the distant metastasis of pancreatic tumors in vivo [61]. Huang et al. [62] treated macrophages with thapsigargin or tunicamycin to induce the ER stress response and observed an upregulation of TNFSF10 expression. In a separate study by Jiang et al. [63], it was noted that TRAIL facilitates cytokine expression and activates the ER stress-dependent NF-κB pathway. These findings collectively suggest a close association between TNFSF10 and ER stress. Superoxide dismutase 2 (SOD2) is an enzyme that plays an important role in reactive oxygen species (ROS) signaling. The study found that silencing SOD2 significantly reduced the growth and metastasis characteristics of PDAC such as migration and colony-forming ability [64]. Moreover, inhibiting SOD2 significantly promotes ER stress, indicating that SOD2 suppression may further enhance endoplasmic reticulum stress by promoting oxidative stress [65]. Prolyl 4-hydroxylase, β polypeptide (P4HB) is an endoplasmic reticulum molecular chaperone protein with oxidoreductase activity. Although downregulation of P4HB was observed in the bulk RNA-seq dataset, its upregulation in the single-cell transcriptome dataset was evident. Furthermore, the experimental results in this study demonstrated that high P4HB expression was correlated with adverse prognosis in PDAC. The highly active interaction between LGALS9 and P4HB also suggested that they played a crucial role in promoting PDAC [66].

The immune landscape of high- and low-risk groups showed that the infiltration level of anti-tumor immune cells such as CD8^+^ T cells, M1 macrophages and NK cells, as well as regulatory T cells were decreased in high-risk group, while activated CD4^+^ T cells and Th2 CD4^+^ T cells were up-regulated in high-risk group. This suggests that the changes of immune infiltrating cells in the PDAC tumor microenvironment are very complex, including the decrease of anti-tumor related immune cells, the increase of negative immunomodulatory cells, and the increase of positive immune cells. Through the analysis of immune infiltrating cells in the tumor microenvironment and the analysis of immune function, we found that the immunosuppressive influence was more obvious in the high-risk group. By further investigating intercellular communication in the high- and low-ERS groups, we found that in the high-ERS group, epithelial cells’ interactions with monocytes, macrophages, and T cells decreased, but the overall number and intensity of intercellular communication increased, including three interesting signaling pathways. These are the MIF, APP and SPP1 signaling pathways. The MIF receptor consists of a ligand-binding CD74 signaling complex coupled with a signal transduction CD44. When ligands bind to MIF, downstream signal transduction can be initiated to promote inflammatory and cell survival [67]. APP may promote the growth of pancreatic cancer cells through the signaling of sAPP and serve as a new therapeutic target for pancreatic cancer [68]. Enrichment of SPP1^+^ macrophages in tumor tissues is negatively correlated with lymphocyte infiltration, indicating poor survival and resistance to immunotherapy [69]. This suggested that complex cell–cell communication can activate downstream signaling pathways in the tumor microenvironment, thus playing an important role in tumor progression. Furthermore, in the liver metastasis group, a notable decrease in taurine metabolism was observed in T cells. Studies have indicated that augmenting taurine levels can enhance effector and memory T cell responses, while inhibiting taurine uptake may induce T cell death [70, 71]. These findings align with our research results, suggesting a phenomenon of T cell immune tolerance mediated by metabolic reshaping during the pancreatic cancer liver metastasis process. There was a significant upregulation in taurine and hypotaurine metabolism in the high-ERS subgroup of primary tumors, while reversed in the liver metastatic cells. Taurine is known for its roles in maintaining the normal electron transport chain, enhancing antioxidant responses, increasing membrane stability, and preventing calcium accumulation [72]. These results indicated that adaptive changes with ER stress and homeostatic imbalances may occur within tumor cells during the process of pancreatic cancer liver metastasis.

## Conclusion

Collectively, our study constructed a novel prognostic model based on machine learning methods to explore and elucidate the important effects of ER stress and lipid metabolism on pancreatic cancer prognosis, immune microenvironment, and metabolism.

### Supplementary Information

**Additional file 1: Figure S1. **Flow chart of steps followed for data collection and analysis in the study. **Figure S2. **Effects of endoplasmic reticulum stress and lipid metabolism on prognosis of pancreatic cancer patients and identification of differentially expressed genes in pancreatic cancer. **A** NMF clustering of pancreatic patients based on ER stress genes in TCGA cohort. **B** Kaplan–Meier curve of OS time in cluster1 and cluster2. **C** Enrichment analysis of cancer related hallmarks in cluster1 and cluster2. **D** NMF clustering of pancreatic patients based on lipid metastatic genes in TCGA cohort. **E** Kaplan–Meier curve of OS time in cluster1 and cluster2. **F** Enrichment analysis of cancer related hallmarks in cluster1 and cluster2. **G, H** Differentially expression analysis between normal and tumor tissues in combined cohorts of TCGA and GTEx. |logFC| ＞ 1 & FDR ＜ 0.05. **Figure S3. **Identification of liver metastatic genes. **A** Cell score heatmap based on SingleR package in GSE197177 data set. **B, C** Cell clustering based on TSNE and UMAP methods and cell annotation in GSE197177. **D** GSVA analysis of hallmarks in cell populations of primary group. **E** GSVA analysis of hallmarks in cell populations of liver metastatic group. **F, G** Enhanced volcano maps of differentially expression analyses in GSE154778 and GSE197177 data sets (FDR ＜ 0.05). **H, I** Volcano maps of differentially expression analyses in GSE71729 and GSE34153 data sets (|log FC| ＞ 1 & FDR ＜ 0.05). **J, K, L** Venn maps showing the screening of liver metastatic genes. **Figure S4. **Evaluation of the prognostic model. Univariate cox regression analyses (left) and the multivariate cox regression analyses (right) of risk score and clinical characteristics in the **A, B** test set, **C, D** TCGA set, **E, F** ICGC set. Risk score of the prognostic model can predict the survival time of patients in subgroups stratified by **G, H** Age, **I, J** Gender, **K, L** Grade, **M, N** T, **O, P** M, and **Q, R** N. **Figure S5. **Correlation of amino acid metabolism related genes with cancer hallmark signaling pathways. **A–C** The correlation of key signaling pathway characteristics with risk genes including SOD2, TNFSF10 and P4HB, as well as **D–G** protective genes including NPC1L1, ADH1C, RAP1GAP and APOE. The color gradient signifies the varying strength of correlation, with dashed lines denoting negative correlations and solid lines representing positive correlations. In terms of statistical significance, shades of colors are employed: cyan corresponds to *p* < 0.001, orange to *p* < 0.01, purple to *p* < 0.05, pink indicating lack of practical significance, and green signifying *p* > 0.05. **Figure S6. **Immune infiltration analyses between high- and low-risk groups. **A–C** CIBERSORT analysis of the immune infiltration levels in high- and low-risk groups in the train set. **D** Correlation analyses of risk score in TCGA cohort with the infiltrating levels of immune cells from XCELL, TIMER, QUANTISEQ, MCPCOUNTER, EPIC, CIBERSORT-ABS and CIBERSORT. **E** Lolipop maps displaying the relationship of immune cells with the 7 selected model genes. **Figure S7. **Cell-cell communication analysis between high- and low-lipid metabolism groups. **A** The number and strength of cell interaction pathways in high- and low-lipid metabolism groups. **B** Circle plots of communicating number and strength between different cell populations. **C** The ranking bar chart showed the signal axes of interactive networks in high- and low-lipid metabolism groups. **D** Heatmap of cell–cell communication number and strength. **E** Bubble map of altered cell-cell communication mediated by individual signaling axes. **F** Heatmaps displayed the overall (comprising of outgoing and incoming) signal flows of each cell subgroup. **Figure S8. **Gene expression and survival analyses. **A** The differentially expression analyses of 7 selected model genes in the high- and low-risk groups. **B** Kaplan–Meier curves of OS of 7 selected model genes in the train set.

**Additional file 2: Table S1**. The information of datasets in this study.

**Additional file 3: Table S2.** Primer sequences in qPCR.

**Additional file 4: Table S3.** The results of Univariate cox regression analysis.

**Additional file 5. Table S4.** The results of GSEA analysis between high- and low- risk groups.

**Additional file 6. Table S5.** The results of subclass mapping for immunotherapy response.

## Data Availability

The datasets analyzed during the current study are available in public databases such as TCGA (https://portal.gdc.cancer.gov/), GEO (https://www.ncbi.nlm.nih.gov/geo/) and ICGC(https://dcc.icgc.org/). The source code can be obtained from GitHub (https://github.com/liu-xiaohong/supplementary_codes).

## Abbreviations

PDAC: Pancreatic ductal adenocarcinoma
ER: Endoplasmic reticulum
OS: Overall survival
TME: Tumor microenvironment
TIDE: Tumor immune dysfunction and exclusion
IPS: Immune cell proportion score
WGCNA: Weighted correlation network analysis
ROC: Receiver operating characteristic
AUC: Area under the ROC curve
DCA: Decision curve analysis
LOOCV: Leave-one-out cross-validation
GSEA: Gene set enrichment analysis
GSVA: Gene set variation analysis
PFS: Progress free survival
