# Supplementary material for: Comprehensive analysis of bulk and single-cell transcriptomic data reveals a novel signature associated with endoplasmic reticulum stress, lipid metabolism, and liver metastasis in pancreatic cancer
Source: J Transl Med. 2024 Apr 29;22:393. doi: 10.1186/s12967-024-05158-y (PMC11057100; doi:10.1186/s12967-024-05158-y)

Supplementary Materials

Comprehensive Analysis of Bulk and Single-Cell Transcriptomic Data Reveals a Novel signature Associated with Endoplasmic Reticulum Stress, Lipid Metabolism, and Liver Metastasis in Pancreatic Cancer

Xiaohong Liu[123#], Bo Ren[123#], Yuan Fang[123#], Jie Ren[123], Xing Wang[123], Minzhi Gu[123], Feihan Zhou[123], Ruiling Xiao[123], Xiyuan Luo[123], Lei You[123*], Yupei Zhao[123*]

[1] Department of General Surgery, Peking Union Medical College Hospital, Peking Union Medical College, Chinese Academy of Medical Sciences, Beijing, 100023, P.R. China.

[2] Key Laboratory of Research in Pancreatic Tumor, Chinese Academy of Medical Sciences, Beijing, 100023, P.R. China.

[3] National Science and Technology Key Infrastructure on Translational Medicine in Peking Union Medical College Hospital, Beijing, 100023, P.R. China.

***Correspondence: Lei You and Yupei Zhao, Department of General Surgery, Chinese Academy of Medical Sciences, Peking Union Medical College, Peking Union Medical College Hospital, Beijing, 100023, People's Republic of China. Email: florayo@163.com; zhao8028@263.net.**

#Xiaohong Liu, Bo Ren and Yuan Fang contributed equally.

Figure S1. Flow chart of steps followed for data collection and analysis in the study.

Figure S2. Effects of endoplasmic reticulum stress and lipid metabolism on prognosis of pancreatic cancer patients and identification of differentially expressed genes in pancreatic cancer. (A) NMF clustering of pancreatic patients based on ER stress genes in TCGA cohort. (B) Kaplan-Meier curve of OS time in cluster1 and cluster2. (C) Enrichment analysis of cancer related hallmarks in cluster1 and cluster2. (D) NMF clustering of pancreatic patients based on lipid metastatic genes in TCGA cohort. (E) Kaplan-Meier curve of OS time in cluster1 and cluster2. (F) Enrichment analysis of cancer related hallmarks in cluster1 and cluster2. (G,H) Differentially expression analysis between normal and tumor tissues in combined cohorts of TCGA and GTEx. $|logFC| > 1$& FDR < 0.05.

Figure S3. Identification of liver metastatic genes. (A) Cell score heatmap based on SingleR package in GSE197177 data set. (B,C) Cell clustering based on TSNE and UMAP methods and cell annotation in GSE197177. (D) GSVA analysis of hallmarks in cell populations of primary group. (E) GSVA analysis of hallmarks in cell populations of liver metastatic group. (F, G) Enhanced volcano maps of differentially expression analyses in GSE154778 and GSE197177 data sets (FDR < 0.05). (H, I) Volcano maps of differentially expression analyses in GSE71729 and GSE34153 data sets ($|log FC| > 1$ & FDR < 0.05). (J,K,L) Venn maps showing the screening of liver metastatic genes.

Figure S4. Evaluation of the prognostic model. Univariate cox regression analyses (left) and the multivariate cox regression analyses (right) of risk score and clinical characteristics in the (A,B)test set, (C,D) TCGA set, (E,F) ICGC set. Risk score of the prognostic model can predict the survival time of patients in subgroups stratified by (G,H) Age, (I,J) Gender, (K,L) Grade, (M,N) T, (O,P) M, and (Q,R) N.

Figure S5. Correlation of amino acid metabolism related genes with cancer hallmark signaling pathways. (A-C)The correlation of key signaling pathway characteristics with risk genes including SOD2, TNFSF10 and P4HB, as well as (D-G) protective genes including NPC1L1, ADH1C, RAP1GAP and APOE. The color gradient signifies the varying strength of correlation, with dashed lines denoting negative correlations and solid lines representing positive correlations. In terms of statistical significance, shades of colors are employed: cyan corresponds to $p<0.001$, orange to $p<0.01$, purple to $p<0.05$, pink indicating lack of practical significance, and green signifying $p>0.05$.

Figure S6. Immune infiltration analyses between high- and low-risk groups. (A-C) CIBERSORT analysis of the immune infiltration levels in high- and low-risk groups in the train set. (D) Correlation analyses of risk score in TCGA cohort with the infiltrating levels of immune cells from XCELL, TIMER, QUANTISEQ, MCPCOUNTER, EPIC, CIBERSORT-ABS and CIBERSORT. (E) Lolipop maps displaying the relationship of immune cells with the 7 selected model genes.

Figure S7. Cell-cell communication analysis between high- and low-lipid metabolism groups. (A) The number and strength of cell interaction pathways in high- and low-lipid metabolism groups. (B) Circle plots of communicating number and strength between different cell populations. (C) The ranking bar chart showed the signal axes of interactive networks in high- and low-lipid metabolism groups. (D) Heatmap of cell-cell communication number and strength. (E) Bubble map of altered cell-cell communication mediated by individual signaling axes.(F) Heatmaps displayed the overall (comprising of outgoing and incoming) signal flows of each cell subgroup.

Figure S8. Gene expression and survival analyses. (A) The differentially expression analyses of 7 selected model genes in the high- and low-risk groups. (B) Kaplan-Meier curves of OS of 7 selected model genes in the train set.

Figure S1. Flow chart of steps followed for data collection and analysis in the study.

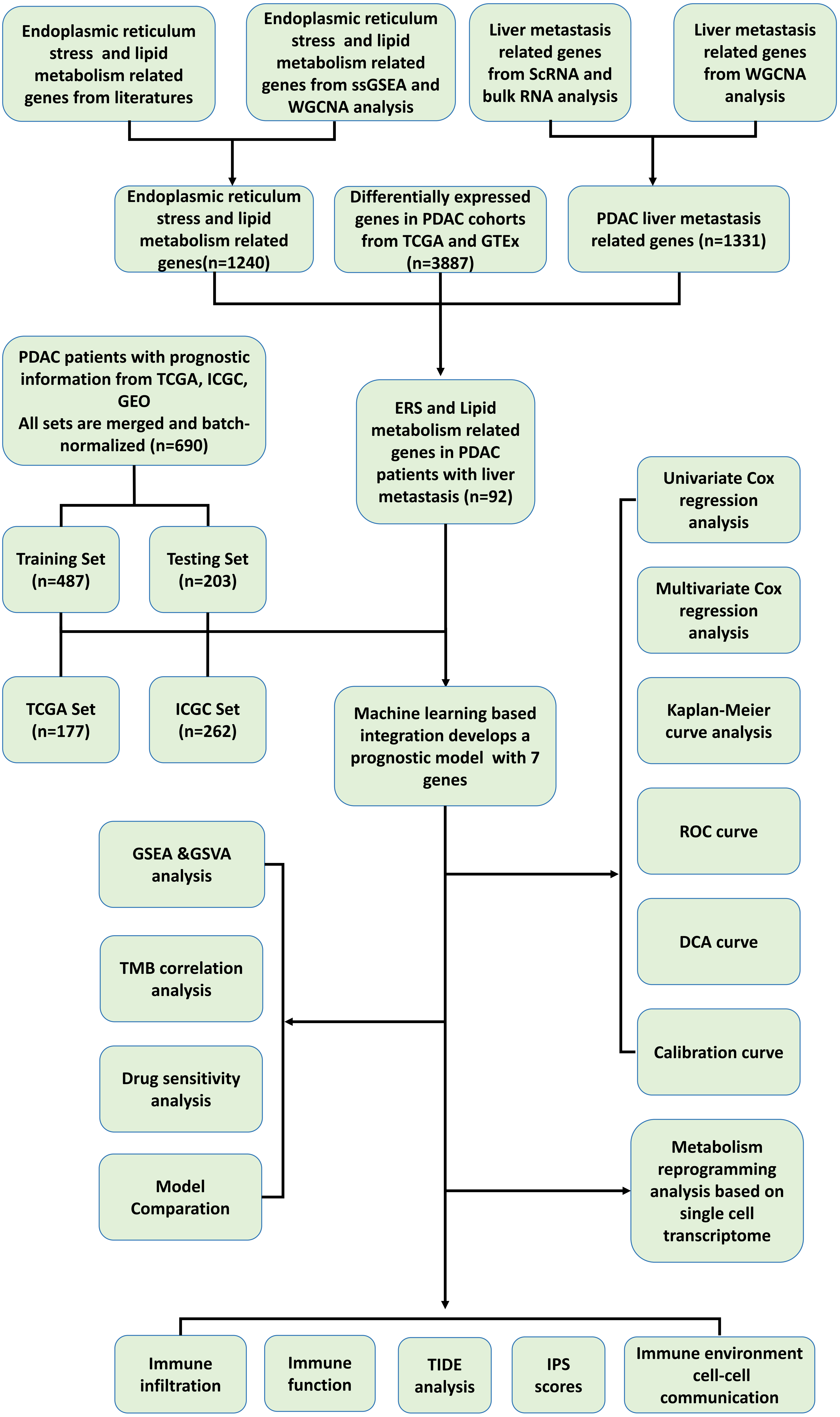

Figure S2. Effects of endoplasmic reticulum stress and lipid metabolism on prognosis of pancreatic cancer patients and identification of differentially expressed genes in pancreatic cancer.

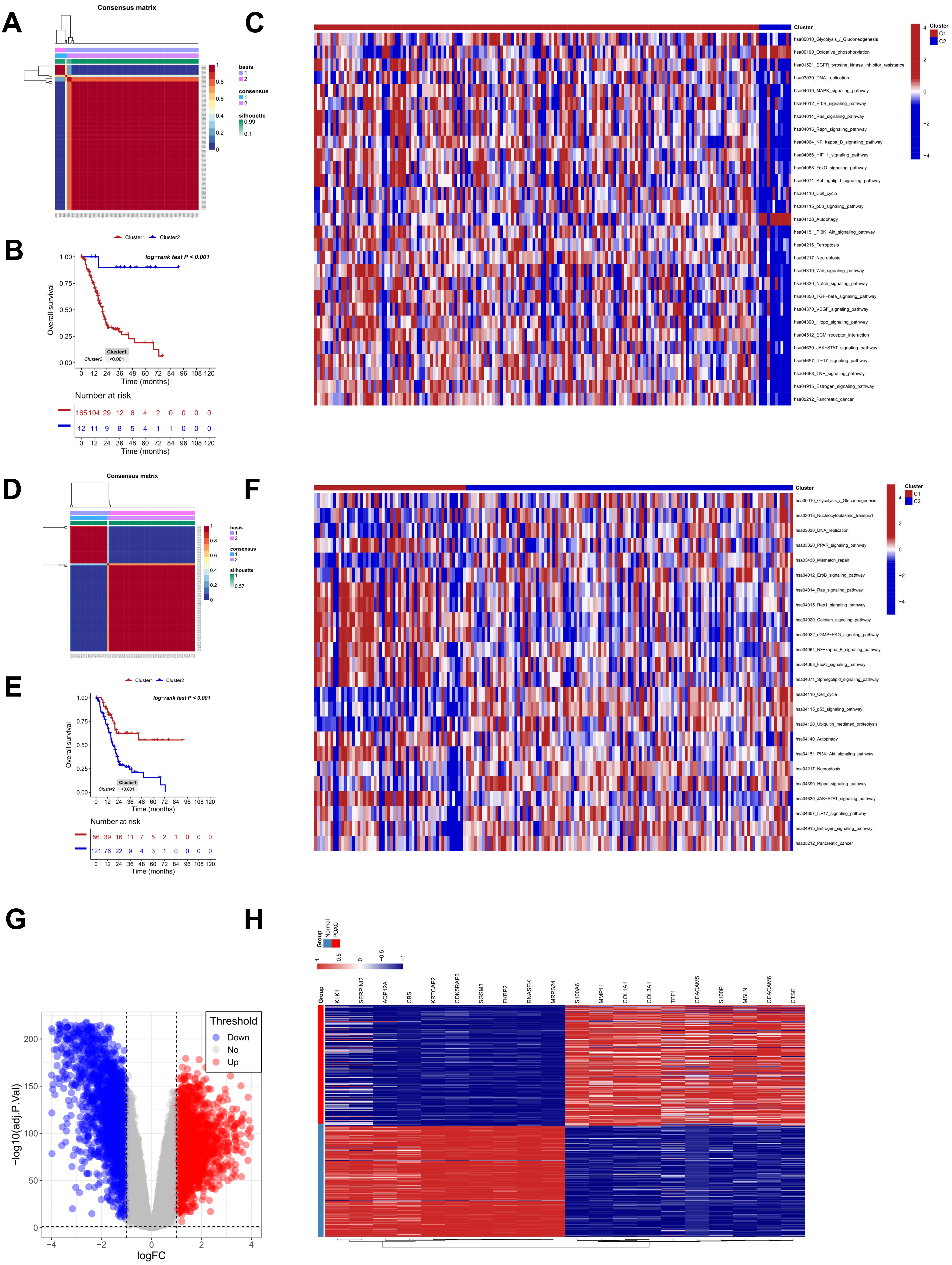

Figure S3. Identification of liver metastatic genes.

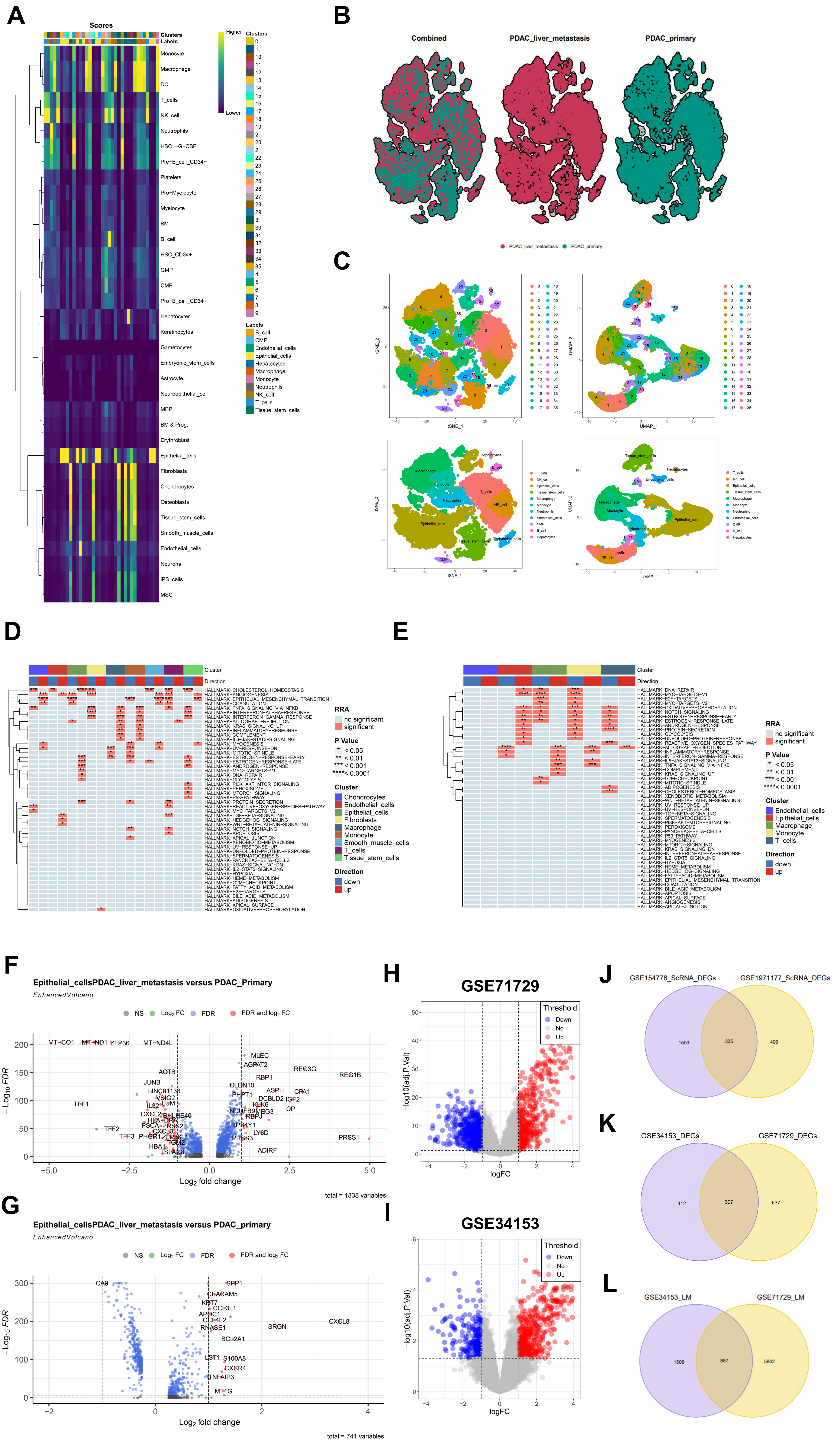

Figure S4. Evaluation of the prognostic model.

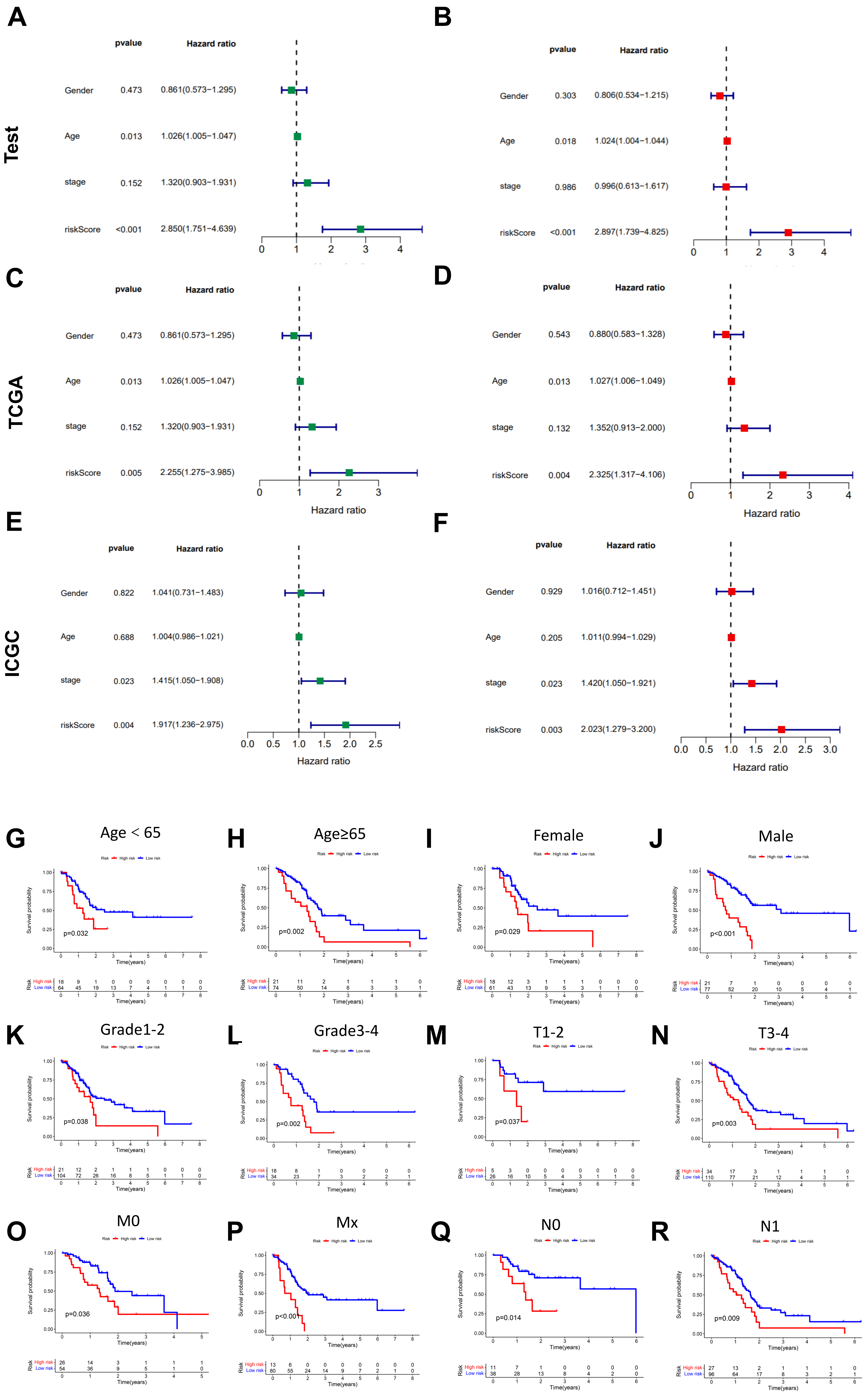

Figure S5. Correlation of amino acid metabolism related genes with cancer hallmark signaling pathways.

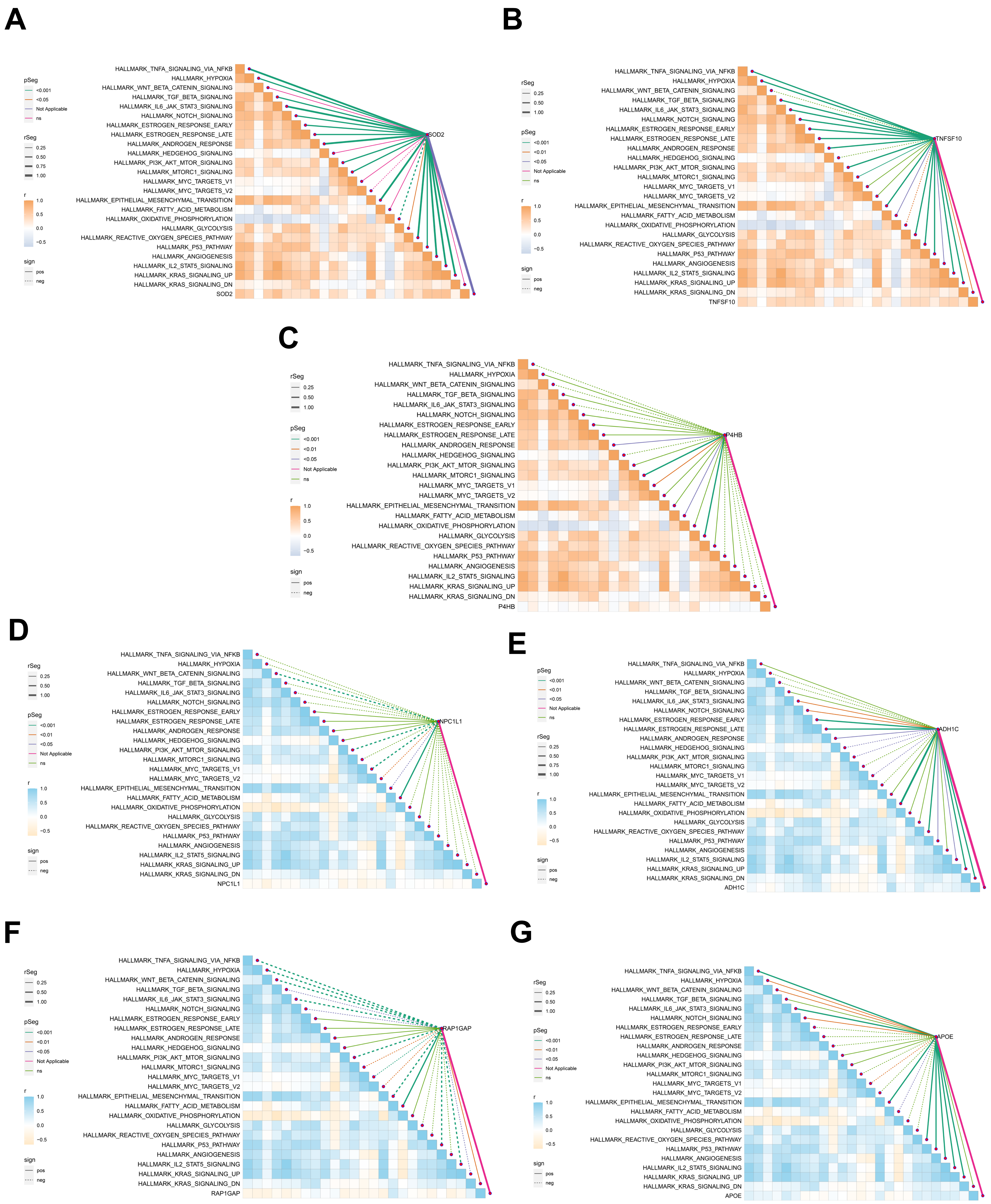

Figure S6. Immune infiltration analyses between high- and low-risk groups.

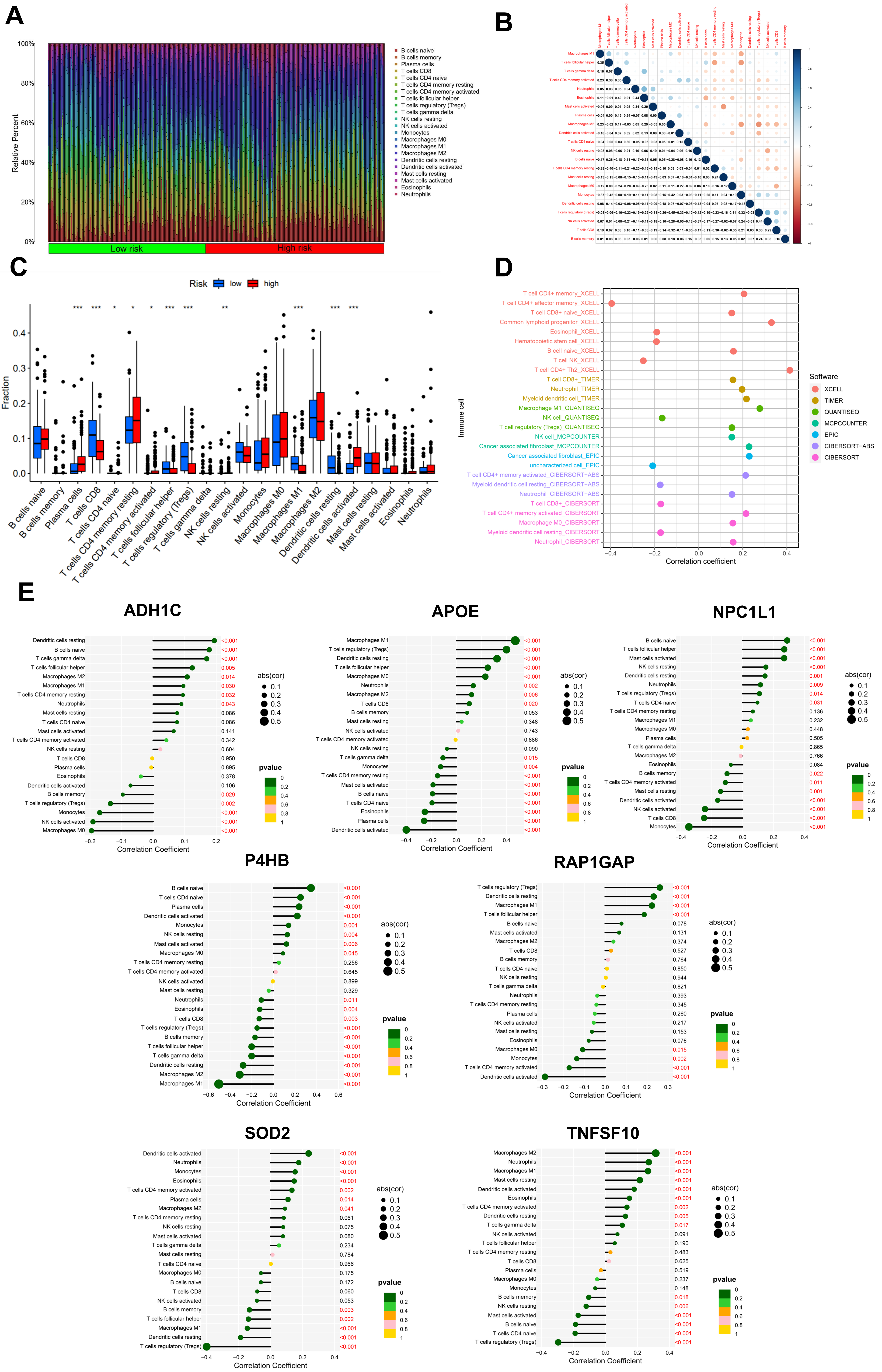

Figure S7. Cell-cell communication analysis between high- and low-lipid metabolism groups

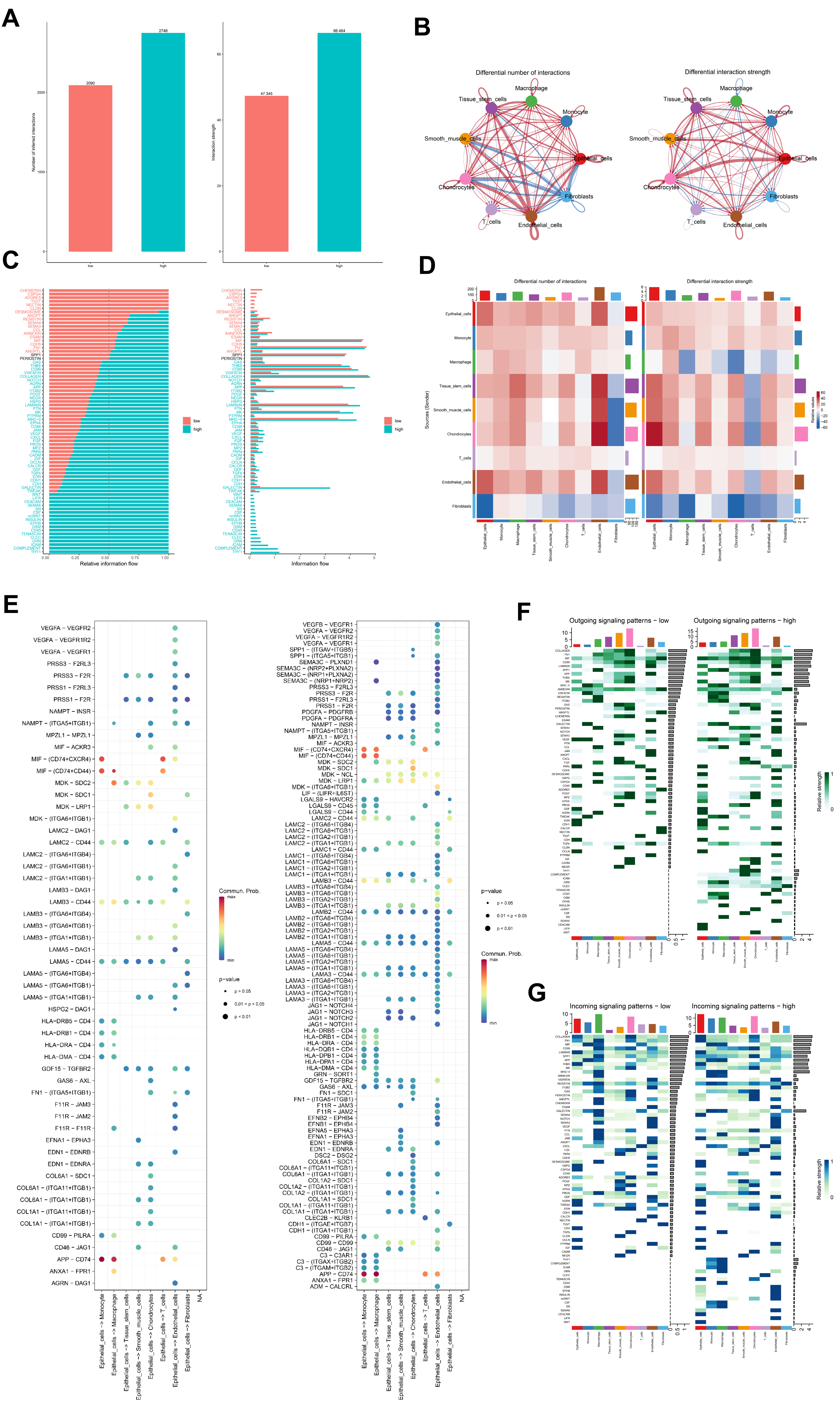

Figure S8. Gene expression and survival analyses.

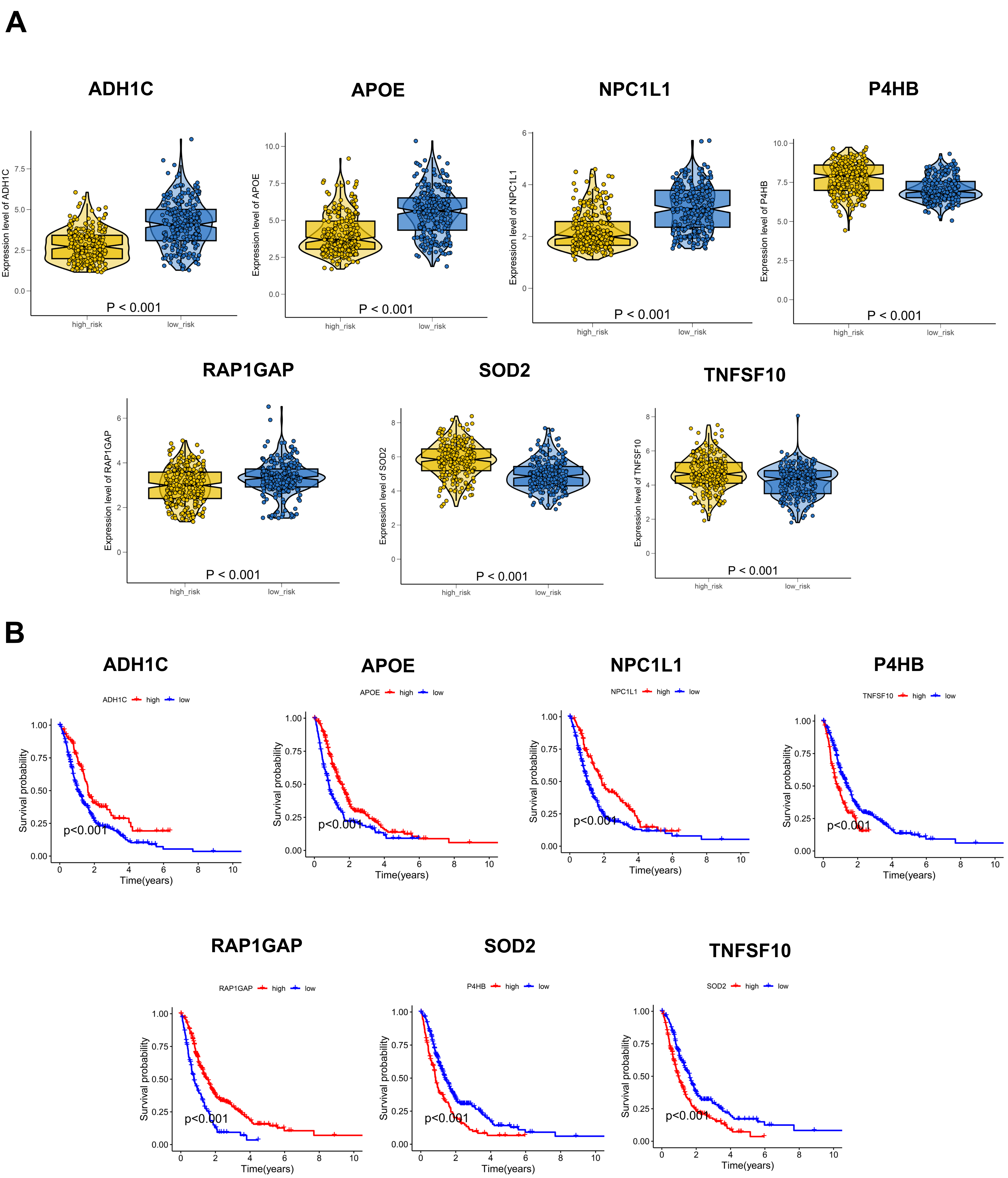

Supplement: Supplementary file 1 — Additional file 1: Figure S1. Flow chart of steps followed for data collection and analysis in the study. Figure S2. Effects of endoplasmic reticulum stress and lipid metabolism on prognosis of pancreatic cancer patients and identification of differentially expressed genes in pancreatic cancer. A NMF clustering of pancreatic patients based on ER stress genes in TCGA cohort. B Kaplan–Meier curve of OS time in cluster1 and cluster2. C Enrichment analysis of cancer related hallmarks in cluster1 and cluster2. D NMF clustering of pancreatic patients based on lipid metastatic genes in TCGA cohort. E Kaplan–Meier curve of OS time in cluster1 and cluster2. F Enrichment analysis of cancer related hallmarks in cluster1 and cluster2. G, H Differentially expression analysis between normal and tumor tissues in combined cohorts of TCGA and GTEx. |logFC| ＞ 1 & FDR ＜ 0.05. Figure S3. Identification of liver metastatic genes. A Cell score heatmap based on SingleR package in GSE197177 data set. B, C Cell clustering based on TSNE and UMAP methods and cell annotation in GSE197177. D GSVA analysis of hallmarks in cell populations of primary group. E GSVA analysis of hallmarks in cell populations of liver metastatic group. F, G Enhanced volcano maps of differentially expression analyses in GSE154778 and GSE197177 data sets (FDR ＜ 0.05). H, I Volcano maps of differentially expression analyses in GSE71729 and GSE34153 data sets (|log FC| ＞ 1 & FDR ＜ 0.05). J, K, L Venn maps showing the screening of liver metastatic genes. Figure S4. Evaluation of the prognostic model. Univariate cox regression analyses (left) and the multivariate cox regression analyses (right) of risk score and clinical characteristics in the A, B test set, C, D TCGA set, E, F ICGC set. Risk score of the prognostic model can predict the survival time of patients in subgroups stratified by G, H Age, I, J Gender, K, L Grade, M, N T, O, P M, and Q, R N. Figure S5. Correlation of amino acid metabolism related genes with cance [file 12967_2024_5158_MOESM1_ESM.pdf]
