# Supplementary material for: Comprehensive analysis of bulk and single-cell transcriptomic data reveals a novel signature associated with endoplasmic reticulum stress, lipid metabolism, and liver metastasis in pancreatic cancer
Source: J Transl Med. 2024 Apr 29;22:393. doi: 10.1186/s12967-024-05158-y (PMC11057100; doi:10.1186/s12967-024-05158-y)
Supplement: Supplementary file 2 — Additional file 2: Table S1. The information of datasets in this study. [file 12967_2024_5158_MOESM2_ESM.docx]

| Table S1. The information of datasets in this study | | | |
| --- | --- | --- | --- |
| Database | Dataset | Description |  |
| TCGA | PAAD | 179 tumor samples, 4 non-tumor samples | bulk transcriptome |
| GTEx | Pancreas | 167 non-tumor samples | bulk transcriptome |
| ICGC | PA_CA | 195 tumor samples | bulk transcriptome |
|  | PA_AU | 82 tumor samples | bulk transcriptome |
| GEO | GSE71729 | 145 primary PDAC samples and 25 liver metastasis samples | bulk transcriptome |
|  | GSE34153 | 14 primary tumor samples and 20 liver metastatic samples | bulk transcriptome |
|  | GSE28735 | 45 tumor samples and 45 non-tumor samples | bulk transcriptome |
|  | GSE57495 | 63 tumor samples | bulk transcriptome |
|  | GSE62452 | 69 tumor samples and 61 non-tumor samples | bulk transcriptome |
|  | GSE85916 | 80 tumor samples | bulk transcriptome |
|  | GSE197177 | 3 primary pancreatic tumor samples and 4 liver metastatic samples | single cell transcriptome |
|  | GSE154778 | 10 primary pancreatic tumor samples and 5 liver metastatic samples | single cell transcriptome |
