# Supplementary material for: Comprehensive analysis of bulk and single-cell transcriptomic data reveals a novel signature associated with endoplasmic reticulum stress, lipid metabolism, and liver metastasis in pancreatic cancer
Source: J Transl Med. 2024 Apr 29;22:393. doi: 10.1186/s12967-024-05158-y (PMC11057100; doi:10.1186/s12967-024-05158-y)
Supplement: Supplementary file 3 — Additional file 3: Table S2. Primer sequences in qPCR. [file 12967_2024_5158_MOESM3_ESM.docx]

Table S2. Primer sequences in qPCR.

| Gene | Forward primer (5'-3') |  | Reverse primer (5'-3') |
| --- | --- | --- | --- |
| GAPDH | ACCCACTCCTCCACCTTT |  | CTGTTGCTGTAGCCAAATTCGT |
| SOD2 | GTTCAATGGTGGTGGTCATATCA |  | GCAACTCCCCTTTGGGTTCT |
| P4HB | TCACCAAGGAGAACCTACTGGA |  | GGCAAGAACAGCAGGATGTGAG |
| TNFSF10 | GACCTGCGTGCTGATCGTGATC |  | GCTGACGGAGTTGCCACTTGAC |
