# Supplementary material for: Comprehensive analysis of bulk and single-cell transcriptomic data reveals a novel signature associated with endoplasmic reticulum stress, lipid metabolism, and liver metastasis in pancreatic cancer
Source: J Transl Med. 2024 Apr 29;22:393. doi: 10.1186/s12967-024-05158-y (PMC11057100; doi:10.1186/s12967-024-05158-y)
Supplement: Supplementary file 4 — Additional file 4: Table S3. The results of Univariate cox regression analysis. [file 12967_2024_5158_MOESM4_ESM.docx]

Table S3. The results of Univariate cox regression analysis.

| Gene |  | HR |  | HR.95L |  | HR.95H |  | P_value |
| --- | --- | --- | --- | --- | --- | --- | --- | --- |
| AGR2 |  | 0.916769 |  | 0.881966 |  | 0.952945 |  | 1.08E-05 |
| CEBPB |  | 0.918992 |  | 0.868933 |  | 0.971934 |  | 0.003116 |
| PPP1R15A | | 0.801292 |  | 0.742394 |  | 0.864862 |  | 1.29E-08 |
| P4HB |  | 1.257292 |  | 1.137864 |  | 1.389255 |  | 6.92E-06 |
| RCN3 |  | 0.908309 |  | 0.859779 |  | 0.959578 |  | 0.000597 |
| ADH1C |  | 0.915336 |  | 0.858489 |  | 0.975947 |  | 0.006847 |
| ECHS1 |  | 0.879612 |  | 0.808735 |  | 0.956701 |  | 0.002766 |
| CPT2 |  | 0.854269 |  | 0.732405 |  | 0.99641 |  | 0.044881 |
| APOE |  | 0.836443 |  | 0.789441 |  | 0.886244 |  | 1.43E-09 |
| PNLIPRP1 |  | 1.061659 |  | 1.022453 |  | 1.102369 |  | 0.00183 |
| UGCG |  | 1.327372 |  | 1.156294 |  | 1.523761 |  | 5.75E-05 |
| PTGS2 |  | 1.194708 |  | 1.108508 |  | 1.287611 |  | 3.22E-06 |
| SCD |  | 0.917724 |  | 0.842754 |  | 0.999363 |  | 0.048311 |
| NPC1L1 |  | 0.749176 |  | 0.678536 |  | 0.827169 |  | 1.10E-08 |
| FOS |  | 0.9052 |  | 0.839642 |  | 0.975878 |  | 0.009417 |
| MIB2 |  | 0.758065 |  | 0.666969 |  | 0.861604 |  | 2.23E-05 |
| RAC1 |  | 0.851102 |  | 0.783424 |  | 0.924627 |  | 0.000137 |
| SOD2 |  | 1.300533 |  | 1.194541 |  | 1.415929 |  | 1.38E-09 |
| TNFSF10 |  | 1.269336 |  | 1.153542 |  | 1.396754 |  | 1.03E-06 |
| NR5A2 |  | 1.141952 |  | 1.066632 |  | 1.222591 |  | 0.000137 |
| RAP1GAP |  | 0.683317 |  | 0.621219 |  | 0.751623 |  | 4.74E-15 |
| MKNK1 |  | 1.361882 |  | 1.241436 |  | 1.494013 |  | 6.25E-11 |
| PIGR |  | 0.941712 |  | 0.905416 |  | 0.979463 |  | 0.002747 |
| CPA2 |  | 1.032651 |  | 1.004266 |  | 1.061839 |  | 0.023863 |
| PRDX4 |  | 0.767884 |  | 0.698357 |  | 0.844333 |  | 4.92E-08 |
| IMPA2 |  | 0.823721 |  | 0.74146 |  | 0.915108 |  | 0.000303 |
| SLC3A1 |  | 1.075728 |  | 1.019597 |  | 1.134949 |  | 0.007591 |
| CTRB2 |  | 1.029744 |  | 1.005119 |  | 1.054972 |  | 0.017624 |
| CHST4 |  | 0.857894 |  | 0.779046 |  | 0.944722 |  | 0.001833 |
| LAPTM4B |  | 0.864461 |  | 0.754967 |  | 0.989836 |  | 0.035048 |
| MT1H |  | 1.295889 |  | 1.202142 |  | 1.396948 |  | 1.33E-11 |
| TPST2 |  | 1.191988 |  | 1.093677 |  | 1.299136 |  | 6.36E-05 |
| CPB1 |  | 1.02499 |  | 1.000261 |  | 1.050331 |  | 0.047599 |
| CXCL6 |  | 1.124637 |  | 1.032307 |  | 1.225226 |  | 0.0072 |
| SCTR |  | 0.910569 |  | 0.837806 |  | 0.989652 |  | 0.02747 |
| REG1B |  | 1.04075 |  | 1.012275 |  | 1.070027 |  | 0.004774 |
| MT1G |  | 1.079652 |  | 1.036293 |  | 1.124826 |  | 0.000248 |
| C11orf54 |  | 1.202201 |  | 1.107734 |  | 1.304725 |  | 1.03E-05 |
| MT1F |  | 1.186661 |  | 1.115596 |  | 1.262252 |  | 5.58E-08 |
| SERPINI2 |  | 1.077035 |  | 1.01447 |  | 1.143458 |  | 0.01508 |
| RBP1 |  | 1.129706 |  | 1.054663 |  | 1.210089 |  | 0.000506 |
| ACTB |  | 0.893522 |  | 0.85487 |  | 0.933921 |  | 6.04E-07 |
